# Fabry Disease and the Heart: A Comprehensive Review

**DOI:** 10.3390/ijms22094434

**Published:** 2021-04-23

**Authors:** Olga Azevedo, Filipa Cordeiro, Miguel Fernandes Gago, Gabriel Miltenberger-Miltenyi, Catarina Ferreira, Nuno Sousa, Damião Cunha

**Affiliations:** 1 Cardiology Department, Reference Center on Lysosomal Storage Disorders, Hospital Senhora da Oliveira, 4835-044 Guimarães, Portugal; filipabritocordeiro@gmail.com; 2 Life and Health Sciences Research Institute (ICVS), School of Medicine, University of Minho, 4710-057 Braga, Portugal; miguelgago@hospitaldeguimaraes.min-saude.pt (M.F.G.); gmiltenyi@medicina.ulisboa.pt (G.M.-M.); njcsousa@med.uminho.pt (N.S.); damiaojlcunha@gmail.com (D.C.); 3 ICVS/3Bs PT Government Associate Laboratory, 4805-017 Braga/Guimarães, Portugal; 4 Neurology Department, Reference Center on Lysosomal Storage Disorders, Hospital Senhora da Oliveira, 4835-044 Guimarães, Portugal; 5 Genetics Department, Reference Center on Lysosomal Storage Disorders, Hospital Senhora da Oliveira, 4835-044 Guimarães, Portugal; 6 Cardiology Department, Centro Hospitalar Trás-os-Montes e Alto Douro, 5000-508 Vila Real, Portugal; catarina.m.ferreira.cf@gmail.com; 7 Faculdade de Ciências da Saúde, Centro de Investigação em Ciências da Saúde, Universidade da Beira Interior, 6200-506 Covilhã, Portugal

**Keywords:** Fabry disease, heart, cardiomyopathy, enzyme replacement therapy, migalastat

## Abstract

Fabry disease (FD) is an X-linked lysosomal storage disorder caused by mutations of the *GLA* gene that result in a deficiency of the enzymatic activity of α-galactosidase A and consequent accumulation of glycosphingolipids in body fluids and lysosomes of the cells throughout the body. GB3 accumulation occurs in virtually all cardiac cells (cardiomyocytes, conduction system cells, fibroblasts, and endothelial and smooth muscle vascular cells), ultimately leading to ventricular hypertrophy and fibrosis, heart failure, valve disease, angina, dysrhythmias, cardiac conduction abnormalities, and sudden death. Despite available therapies and supportive treatment, cardiac involvement carries a major prognostic impact, representing the main cause of death in FD. In the last years, knowledge has substantially evolved on the pathophysiological mechanisms leading to cardiac damage, the natural history of cardiac manifestations, the late-onset phenotypes with predominant cardiac involvement, the early markers of cardiac damage, the role of multimodality cardiac imaging on the diagnosis, management and follow-up of Fabry patients, and the cardiac efficacy of available therapies. Herein, we provide a comprehensive and integrated review on the cardiac involvement of FD, at the pathophysiological, anatomopathological, laboratory, imaging, and clinical levels, as well as on the diagnosis and management of cardiac manifestations, their supportive treatment, and the cardiac efficacy of specific therapies, such as enzyme replacement therapy and migalastat.

## 1. Fabry Disease Overview

Fabry disease (FD) (OMIM 301500) is a rare lysosomal storage disorder caused by mutations in the *GLA* gene, leading to deficiency of the enzymatic activity of α-galactosidase A. Globotriaosylceramide (GB3) and other neutral glycosphingolipids consequently accumulate in body fluids and lysosomes of cells throughout the body, including in those that are particularly relevant for the disease, such as in the heart (cardiomyocytes, conduction system cells, vascular endothelial and smooth muscle cells, and fibroblasts), kidney (podocytes, tubular, glomerular, mesangial, and interstitial cells), nervous system (neurons in autonomic and posterior root ganglia) and vascular endothelium and smooth muscle [1,2].

*GLA* mutations causing a virtually null enzymatic activity (<5% of the normal mean) are associated with severe and early onset classical phenotypes, which are characterized by the development of clinical manifestations in childhood or adolescence, such as acroparesthesias, neuropathic pain, hypohidrosis, heat, cold and exercise intolerance, cornea *verticillata*, angiokeratomas, gastrointestinal symptoms, and proteinuria. In adulthood, patients may also suffer from cardiomyopathy, heart failure, dysrhythmias, cardiac conduction blocks, renal failure, brain white matter lesions, cerebrovascular events, and sensorineural deafness. In contrast, *GLA* mutations leading to a residual enzymatic activity are associated to attenuated and late-onset phenotypes, which are characterized by the development of cardiac, renal and/or cerebrovascular manifestations in adulthood [2,3,4,5]. In this X-linked disorder, heterozygote females are not merely carriers and their clinical spectrum widely ranges from asymptomatic to full-blown disease as severe as in affected males [6,7].

## 2. Cardiac Involvement in FD

### 2.1. Pathophysiology

FD leads to GB3 accumulation in virtually all cardiac cells, but the mechanisms by which substrate accumulation leads to cellular dysfunction or organ damage remain less defined [8]. GB3 accumulation may affect mitochondrial function, either directly through accumulation within the mitochondrial membrane or indirectly by preventing mitophagy [9], likely contributing to the reduction in the activities of the respiratory chain enzymes that has been shown in fibroblasts [10]. On the other hand, substrate accumulation and organelle damage have also been shown to induce oxidative stress [9,11]. GB3 has also been demonstrated to promote a higher proinflammatory cytokine production and expression [12], to mediate apoptosis [13,14], and to induce endothelial dysfunction [15]. Lyso-GB3, a deacylation product of GB3 [16], has also been shown to inhibit α-galactosidase A activity and to promote the proliferation of smooth muscle cells [17], likely contributing to the increased intima-media thickness.

### 2.2. Pathology 

GB3 deposits are found in cardiomyocytes, valve fibroblasts, endothelial and smooth muscle vascular cells, and cardiac conduction system cells [18,19,20,21], representing 1–2% of the cardiac mass. Nevertheless, in cardiac variants, lysosomal inclusions are only found in cardiomyocytes [22]. In women, a mosaic pattern of normal and vacuolated cells caused by random X-chromosome inactivation is observed [23].

GB3 accumulation activates common signaling pathways leading to hypertrophy, inflammation, apoptosis, necrosis, and fibrosis. Accordingly, anatomopathological analysis of hearts of FD patients have shown hypertrophy of the cardiomyocytes, myocyte apoptosis and necrosis, inflammatory infiltrate, replacement and interstitial fibrosis, valve thickening and vascular intima and media thickening with vascular narrowing [18,21]. Myocardial disarray may be observed, although it is less pronounced than in sarcomeric HCM [21].

The cardiac involvement by FD is progressive. Progressive cardiomyocyte hypertrophy ultimately ends in cell death of enlarged substrate-engorged cardiomyocytes, either by necrosis or apoptosis, which presumably leads to fibrosis. Accordingly, the cardiomyocyte diameter, the lysosomal glycosphingolipid area and the extent of necrosis, apoptosis and fibrosis are all positively correlated with disease severity and age. In the “pre-hypertrophy” stage, the cardiomyocytes are already mildly hypertrophied and contain numerous glycosphingolipid-engorged vacuoles, mostly in the perinuclear zone, while the intramural vessels and interstitium are essentially unaffected, and myocardial fibrosis is not detectable. In moderate hypertrophy, vacuolar areas occupy > 30% of myocytes, and there is increased cell apoptosis and necrosis, moderate fibrosis, and thickening and some luminal narrowing of intramural vessels. In severe hypertrophy, lysosomal inclusions occupy ~60% of the myocardial cells, and there is extensive myocardial fibrosis and severe narrowing of arteriole lumens. Cell apoptosis seem to prevail in patients with moderate cardiac hypertrophy, while myocyte necrosis is more pronounced in severe hypertrophy [23].

Endomyocardial biopsies have shown myocarditis in 56% of FD patients. Myocarditis was detected even before left ventricular hypertrophy (LVH) or late gadolinium enhancement (LGE) and its frequency correlated with disease severity. Myocarditis is immune-mediated and positive antiheart and antimyosin antibodies were found in all FD patients with myocarditis [24]. 

## 3. Cardiac Manifestations of FD

Cardiac manifestations of FD include ventricular hypertrophy and fibrosis, valve thickening or regurgitation, heart failure, angina, dysrhythmias, cardiac conduction abnormalities, and sudden death [25]. Cardiac signs or symptoms have been reported in 60% of males and 50% of females, with a mean age of onset of 29.2 ± 14.4 and 34.5 ± 17.6 years, respectively [26]. Cardiac symptoms were the presenting symptoms of FD in 13% of males and 10% of females [27] (Table 1). Despite available therapies and supportive treatment, cardiac involvement carries a major prognostic impact, representing the main cause of death in FD [26,28].

### 3.1. Hypertrophic Cardiomyopathy

Hypertrophic cardiomyopathy (HCM) is the main cardiac manifestation of FD [25]. FD has been diagnosed in 0.9% of the patients with HCM [29,30] and, being a treatable disease, it should be systematically ruled out in all patients with HCM, either by targeted FD screening or by a wider HCM gene panel including the *GLA* gene [29]. Basal inferolateral LGE and bifascicular block were identified as the most powerful predictors of FD in patients with HCM; therefore, in their presence, a targeted FD screening should be performed, while in their absence, an HCM gene panel would be the most appropriate next step in the etiological study of HCM [29].

#### 3.1.1. Left Ventricular Hypertrophy

LVH was found in 43% of males and 26% of females, arising earlier and progressing more rapidly in males than in females (mean age of onset: 39 ± 10 vs. 50 ± 11 years) [31,32]. The prevalence of LVH has been reported to increase with age, occurring in 76.9% of patients aged ≥ 75 years [33]. Left ventricular (LV) mass index was also found to increase with age [34,35] and to correlate inversely with estimated glomerular filtration rate (eGFR) [34]. In a late-onset phenotype with predominant cardiac involvement, LVH was detected in 73.1% of males and 19.0% of females, with a mean age at diagnosis of 57 ± 10 and 73 ± 8 years, respectively. The frequency and severity of LVH also increased with age [4,5].

Although electrocardiographic changes of LVH may predate imaging evidence of LVH in Fabry patients [35], multimodality cardiac imaging is useful to suggest the diagnosis of FD, to detect LVH, as well as to monitor the progression of cardiac involvement and response to therapy [36,37]. Cardiac magnetic resonance imaging (MRI) can detect cardiac involvement even when the LVH severity is mild, allowing to reclassify 21% of FD patients as having cardiac involvement that was previously unrecognized [38]. Of note, recent studies have suggested that machine learning applied to 3D myocardial architecture and deformation obtained by cardiac MRI may present increased ability to perform differential diagnosis of the cause of HCM [39].

**Figure 1:**
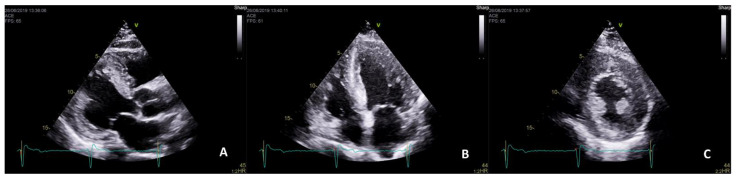
Left ventricular hypertrophy (LVH) secondary to Fabry disease (FD). Transthoracic echocardiogram of a 70-year-old male Fabry patient showing a severe symmetrical LVH with prominent papillary muscles in parasternal long-axis (**A**), four-chambers (**B**) and short-axis (**C**) views.

LVH secondary to FD is most commonly concentric and symmetric [25,32] (Figure 1); however, other patterns can also occur, including asymmetric septal hypertrophy, eccentric hypertrophy, and apical hypertrophy [25,40,41]. 

A binary appearance of the LV endocardial border, known as the “binary sign,” was correlated with a characteristic pattern of glycosphingolipid compartmentalization on histological examination and once thought to represent a hallmark feature of Fabry cardiomyopathy [42]. However, it was later found to be an unreliable marker of Fabry cardiomyopathy, with an estimated sensitivity of 28.0% and a specificity of 80.0%, occurring more commonly in patients with LVH and in a more advanced stage of the disease [43].

Prominent papillary muscles are a characteristic feature of FD [25,44] (Figure 1), while papillary muscles anomalies, such as anterior displacement of the anterolateral papillary muscles or direct insertion of the papillary muscle into the mitral valve, are very specific for HCM [45]. Indeed, the papillary muscle contribution to LV mass was found to be significantly increased in Fabry patients, both with and without LVH, compared to controls [38]. Hence, the inclusion of papillary muscle mass in LV mass calculation is recommended for the earlier detection of LVH in Fabry patients [46], although exclusion of papillary muscles seems to be a better predictor of adverse cardiac events (composite endpoint of ventricular tachycardia (VT), bradycardia requiring device implantation, severe heart failure, and cardiac death) [47]. 

#### 3.1.2. Left Ventricular Storage, Inflammation, and Fibrosis

**Figure 2:**
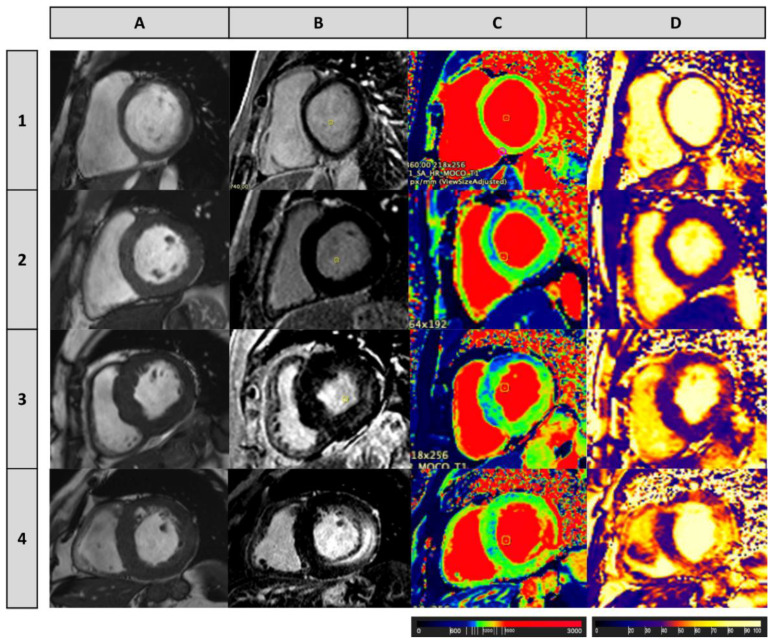
Cardiac magnetic resonance imaging (MRI) (3.0 Tesla) in four Fabry patients, illustrating different stages of myocardial involvement by the disease. (**A**) Cine (balanced steady-state free precession sequence) image at the basal left ventricular (LV) short-axis slice; (**B**) Late gadolinium enhancement (LGE) at the basal LV short-axis slice; (**C**) Native T1 mapping (precontrast) performed using a modified Look-Locker inversion (MOLLI) recovery sequence at the basal LV short-axis slice (the resulting pixel-by-pixel T1 color maps were displayed using a customized lookup table, in which normal myocardium was green, increasing T1 was yellow and red, and decreasing T1 was blue); and (**D**) T2 mapping (fast low angle shot (FLASH)) at basal LV short-axis slice (the resulting pixel-by-pixel T2 color maps were displayed using a customized lookup table, in which normal myocardium was purple and increasing T2 was red and yellow). **Patient 1:** A 26-year-old female without LVH or LGE, presenting normal values of T1 (1265 ± 68 ms at the basal septum) and T2 (41.76 ± 5.40 ms at the basal septum); **Patient 2:** A 41-year-old male without LVH or LGE, presenting low T1 (1118 ± 40 ms) and normal T2 (40.93 ± 5.80 ms) values at the basal septum; **Patient 3:** A 76-year-old female with LVH (LV mass 83 g/m^2^, maximum wall thickness 19 mm at the basal septum) and diffuse LGE in the basal segment of the inferolateral wall, who presents low T1 (1093 ± 36 ms) at the basal septum and T1 pseudonormalization particularly at the inferolateral wall (1276 ± 59 ms), where a mild increase in T2 values (49.10 ± 2.30 ms) was also observed; **Patient 4:** A 69-year-old male patient with LVH (LV mass 123 g/m^2^, maximum wall thickness 18 mm at the septum) and diffuse and extensive LGE in the inferolateral wall, who presents areas of T1 pseudonormalization but also areas of T1 increase, such as in the inferolateral wall (1425 ± 144 ms), where T2 values (64.44 ± 8.56 ms) are also increased.

Intramyocardial LGE on the basal inferolateral LV segments is typically seen on cardiac MRI in approximately 50% of Fabry patients [48] (Figure 2), which correlates histologically with focal replacement fibrosis [49]. This LGE pattern was hypothesized to be due to increased stress at the interface of the fibrous skeleton of the mitral annulus and the LV midwall [50]. It allows to differentiate FD from sarcomeric HCM, which usually results in LGE at the right ventricular (RV) junction points, and amyloidosis, which usually results in a global subendocardial pattern of LGE [51]. However, this LGE pattern is not exclusive of FD and may also be found in myocarditis, Chagas disease, and sarcoidosis [52].

LGE may develop before LVH, and a study has reported that 50% of females would not have been identified with FD cardiomyopathy without the aid of LGE imaging [53]. The prevalence of FD in patients with unexplained LGE has been reported as 2.5% [54].

**Figure 3:**
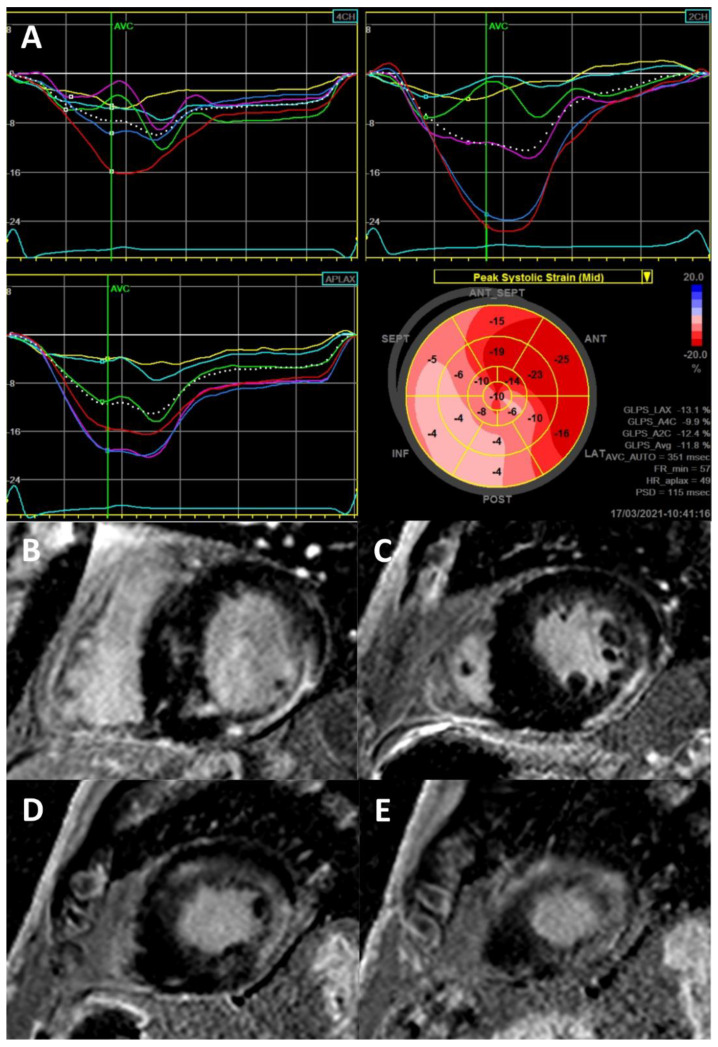
Strain echocardiography for the detection of LGE. (**A**) Transthoracic echocardiogram with 2D-strain analysis by speckle tracking showing reduction of longitudinal strain, particularly in the inferolateral, inferior and inferoseptal walls and apex, in a 73-year-old male Fabry patient; (**B**–**E**) Cardiac MRI (3.0 Tesla) with LGE in short-axis slices, from the LV base to the apex, showing fibrosis in the same regions where the reduction in longitudinal strain was more pronounced.

The “double-peak sign’’ on strain rate by Tissue Doppler Imaging (TDI), depicted as a sharp first peak in early systole followed by a rapid fall of strain rate approaching zero and finally a second peak during isovolumetric relaxation, identifies segments of LGE with 99% of sensitivity and 93% of specificity in patients with HCM, aortic stenosis, and FD [55]. Two-dimensional speckle-tracking can also detect LGE (Figure 3). LV global longitudinal strain (GLS) is lower in patients with LGE and reduced longitudinal systolic strain (worse than −12.5%) in the posterolateral segment identifies LGE with 90% of sensitivity and 97% of specificity [56]. It was suggested that the absence of ST segment or T wave alterations on electrocardiogram could almost exclude LGE in FD [57]; however, although LGE has been significantly associated with ST segment depression and negative T waves, it has been described in up to 17.8% of the patients without ST depression and 13.4% of the patients without negative T waves in a large cohort of patients with late-onset FD with predominant cardiac involvement [5].

In FD patients with basal inferolateral LGE, troponin levels and T2 values are increased, suggesting that LGE may also represent inflammation [58] (Figure 2).

LGE was shown to correlate with increased high-sensitivity troponin, and it has been proposed that normal values of high-sensitivity troponin, in combination with normal ECG and echocardiogram, indicate that FD cardiomyopathy is unlikely; elevated values of high-sensitivity troponin indicate advanced FD cardiomyopathy, while borderline values should lead to a thorough investigation including cardiac MRI. In addition, patients with increased high-sensitivity troponin presented decreased LV wall thickness and ejection fraction, suggesting progression of cardiomyopathy [59]. Increased inflammatory markers, such as interleukin-6 and tumor necrosis factor, were also associated to increased disease burden (LVH and fibrosis) [60].

In another study, LGE in the basal inferolateral wall was associated to high global and basal inferolateral wall T2. High basal inferolateral wall T2 was, in turn, associated with increased troponin and N-terminal prohormone of brain natriuretic peptide (NT-proBNP), GLS impairment, and electrocardiographic abnormalities (long PR, complete bundle branch block, LVH voltage criteria, long QTc, and T-wave inversion) and predicted clinical worsening after 1 year (Fabry stabilization index > 20%) [61].

Finally, LGE regions also showed focal 18F-FDG uptake, further supporting the notion that LGE represents inflammation [62]. Focal 18F-FDG uptake was also shown before the development of LGE in FD females, in association to a pseudonormalization of T1 time, suggesting an intermediate stage of Fabry cardiomyopathy [63]. Focal 18F-FDG uptake was also shown in 50% of the patients before LVH, being associated with lower LV GLS [64].

LGE at the time of initiation of enzyme replacement therapy (ERT) was associated to no improvement in LV mass, LV strain, and exercise capacity [65]. Moreover, LGE was associated with a higher risk of ventricular arrhythmias and sudden cardiac death (SCD) [66,67] and the annual increase in fibrosis was the only independent predictor of ventricular arrhythmias [66]. 

FD has also been associated to low native T1 time on cardiac MRI [68] (Figure 2), which has been attributed to intracellular glycosphingolipid accumulation [68]. In FD patients without LVH, low T1 was found in 40% of the cases [68,69] (Figure 2), being associated with ECG abnormalities and worse LV wall thickness and mass [69,70], LA size [70,71], E/E’ ratio, LV GLS and inferolateral longitudinal strain [71], LV GLS by feature tracking [72], LGE [69], and worse Mainz Severity Score Index (MSSI) and clinical worsening (Fabry Stabilization Index > 20%) [70]. In FD with LVH, T1 correlated inversely with LV mass [68]. In patients with LVH, low native T1 time accurately differentiates FD from other causes of LVH [68], as low T1 times are only otherwise noted in iron overload [73]. Pseudonormalization or elevation of T1 in LV inferolateral wall was correlated with the presence of LGE [68] (Figure 2). In FD patients with RV hypertrophy, low T1 was also found in the RV [74].

Nordin et al. proposed three stages of cardiac involvement in FD: (1) storage stage with normal or low native T1 times without LVH; (2) inflammation and myocyte hypertrophy stage with low native T1 times, inflammation, LGE, chronic troponin elevation, with LVH in males and without LVH in females; and (3) fibrosis and impairment stage with pseudonormalization of native T1 times, extensive LGE, LVH, troponin and NT-proBNP elevation, LV dysfunction, and heart failure [75].

**Figure 4:**
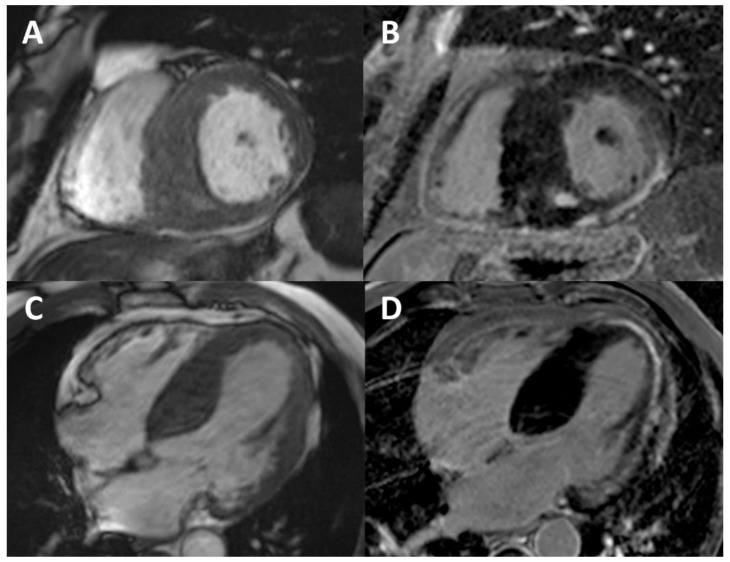
Cardiac MRI (3.0 Tesla) in a Fabry patient with advanced cardiomyopathy. Cine (balanced steady-state free precession sequence) images at the basal LV short-axis slice (**A**) and four-chamber view (**C**) showing massive and asymmetrical LVH (maximal thickness 30. mm at the septum) with thinning of the posterior wall (2 mm). LGE at the basal LV short-axis slice (**B**) and four-chamber view (**D**) showing fibrosis of the inferior and inferolateral walls and apex and focal fibrosis in the septum, where the LVH is more pronounced.

In advanced stages of FD, there is thinning and akinesia of the basal posterior wall, which may result in asymmetrical LVH [25] (Figure 4) and correlates histologically with fibrosis [22]. The thinning of the basal posterior wall was found to significantly precede severe heart failure [New York Heart Association (NYHA) class III] and cardiac death—LV septum/posterior wall thickness ratio >1.3, 1.5, or 1.7 significantly preceded NYHA class III heart failure and cardiac death by 4.0, 3.8, or 3.4 and 4.7, 4.5, or 4.1 years, respectively [76].

#### 3.1.3. Left Ventricular Function

Diastolic function is abnormal in 69.4% of those with LVH [4] and 63% of those with LGE [77]. Diastolic dysfunction occurs more commonly as an abnormal relaxation or a pseudonormal pattern [25], and it has been associated with the presence of LGE [78] and to correlate with NT-proBNP [79]. LV systolic dysfunction with reduction in ejection fraction is rare (6.7%) [80], occurring in late stages of advanced Fabry cardiomyopathy [22,25]. 

LV systolic and diastolic dysfunction can, however, be detected before the development of LVH, not only by TDI [81] but also by speckle-tracking [82,83]. LV longitudinal [82], circumferential and radial strain [83] were found to be reduced in the pre-hypertrophic stage; basal segmental longitudinal strain is also impaired even when LV wall thickness is normal [84], and the strain rate during isovolumic relaxation and the ratio of transmitral E-wave velocity to the strain rate during isovolumic relaxation have shown to differentiate FD patients from controls regardless of LVH [85].

**Figure 5:**
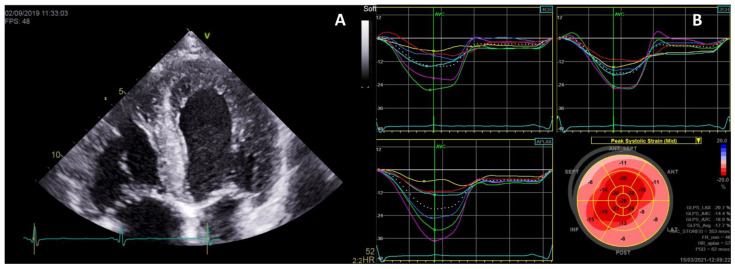
Apical sparing pattern of global longitudinal strain (GLS) in a Fabry patient. Transthoracic echocardiogram of a 48-year-old male Fabry patient showing symmetrical LVH in a four-chamber view (**A**) and 2 D-strain analysis by speckle tracking revealing an apical sparing pattern of GLS (**B**).

Strain echocardiography has also shown that mechanical dispersion is higher in FD patients with LVH than in FD patients without LVH or healthy controls [86]. An apical sparing pattern on longitudinal strain has also been described in FD, similarly to amyloidosis [84,87] (Figure 5). Additionally, the loss of the normal base-to-apex circumferential strain gradient was suggested to be a specific LV deformation pattern of Fabry cardiomyopathy, as opposed to non-obstructive HCM that has been associated to a higher global circumferential strain and a normal base-to-apex gradient [88]. 

#### 3.1.4. Left Ventricular Obstruction

Obstruction at the LV outflow tract (LVOT) may occur, but massive LVH involving the papillary muscles has also been reported to cause mid-ventricular obstruction [89]. Obstruction at rest is rare, but it may be elicited by exercise in 43% of the patients [90], also contributing to heart failure. During exercise, FD patients also present lower augmentation of stroke volume than healthy controls, being E/E’ ratio the independent predictor of exercise-induced change in stroke volume [91].

#### 3.1.5. Right Ventricular Involvement

RV hypertrophy occurs in 31–71% of the patients [92,93]. RV systolic function in patients with Fabry cardiomyopathy tends to be preserved [93]. Nevertheless, RV global and free wall systolic strain may be reduced despite normal function on conventional echocardiography, and RV systolic dysfunction has been associated to RV wall thickness and fibrosis [94]. Although RV hypertrophy and RV systolic function indexes (TAPSE and S’) have shown significant association to clinical events, they were not identified as independent predictors of their occurrence [95].

#### 3.1.6. Atrial Involvement

Glycolipid deposition in the atria [19] may ultimately cause atrial dilation, which occurs more commonly in patients with LVH [81] and fibrosis [56]. Left atrial (LA) systolic and early diastolic strain rate were found preferentially reduced in patients with LVH, but LA systolic strain was decreased even before LVH [96]. Peak atrial longitudinal strain was inversely associated to Fazekas score of brain white matter lesions, even after adjusting for LV mass index [97]. Atrial dilation is associated to the occurrence of atrial fibrillation [98], which also contributes to heart failure.

#### 3.1.7. Heart Failure

Ventricular hypertrophy and fibrosis result in diastolic and systolic dysfunction, which together with dysrhythmias and conduction disorders, valve disease, and myocardial ischemia, contribute to heart failure [25]. Dyspnea or heart failure has been reported in 19.7% of untreated females and 19.4% of untreated males [34]. The prevalence of heart failure increases with age, occurring in 34.6% of the patients aged ≥ 75 years [33]. In a late-onset phenotype with predominant cardiac involvement, heart failure was found in 32.9% of males and 14.8% of females and mean survival free from heart failure was 64 ± 1 and 76 ± 2 years, respectively. The frequency of heart failure also increased with age [4,5].

NT-proBNP levels were correlated to symptom class, LV mass, E/E’ ratio, and LA size, reaching higher values in patients with LVH, diastolic dysfunction, and LGE [75,99].

Severe heart failure (NYHA class ≥ III) was reported in 10% of FD patients. The annual incidence of severe heart failure was 1.62 per 100 person-years, and age and MSSI were independent predictors of its development [100] (Table 2). 

### 3.2. Dysrhythmias and Cardiac Conduction Disorders

As a consequence of dysrhythmias and cardiac conduction disorders, Fabry patients may experience symptoms, such as palpitations and syncope. Palpitations have been reported in 15.3% and 21.3% of untreated Fabry male and female patients, while syncope has been, respectively, reported in 5.6% and 2.4% [34].

#### 3.2.1. Bradycardia, Chronotropic Incompetence, and Cardiac Conduction Disorders

FD involvement of the autonomous nervous system may result in a significantly reduced heart rate variability in pediatric male patients, reflecting a reduction in parasympathetic stimulation of the heart [101].

**Figure 6:**
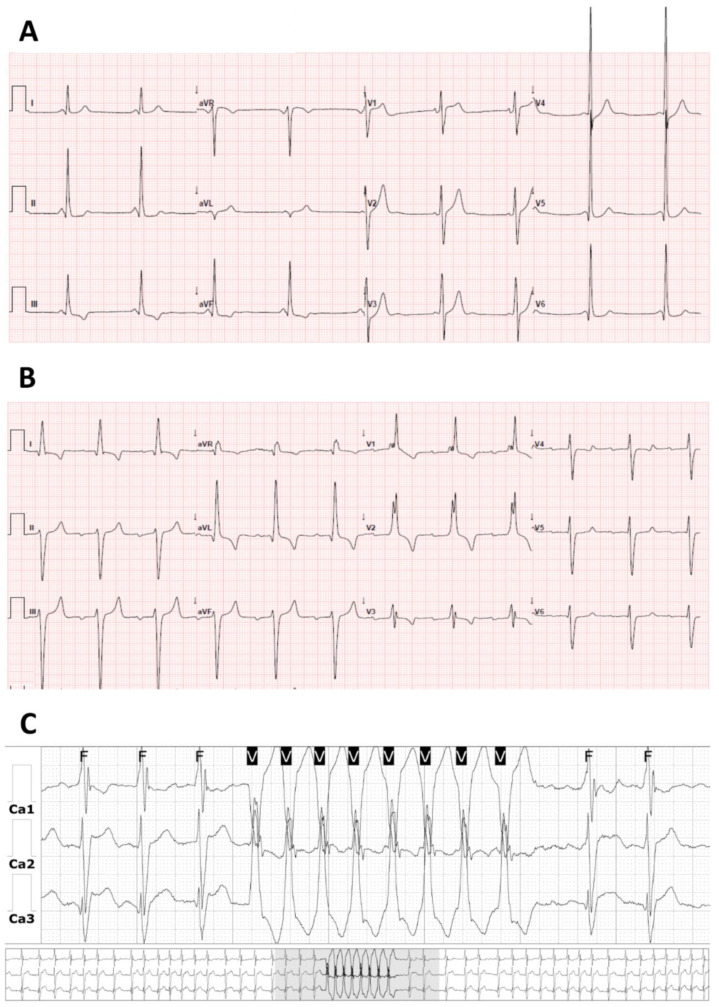
Electrocardiographic findings in FD. (**A**) Short PR interval in a 34-year-old male, (**B**) bifascicular and first-degree AV blocks and electrocardiographic criteria of LVH in a 72-year-old male, and (**C**) non-sustained VT in 24 h-Holter monitoring of a 76-year-old male.

GB3 accumulation in the cardiac conduction system cells [20] is constant in men and variable in women due to X-chromosome inactivation [102]. In an earlier stage, it may lead to accelerated atrioventricular (AV) conduction, which manifests as a short PR interval [102,103] (Figure 6). Although there are reports of FD cases with short PR interval and accessory pathways [104], enhanced AV conduction rather than ventricular pre-excitation is the most likely cause of the short PR interval. Short PR interval has been documented in FD patients in whom pre-excitation due to accessory pathways has been formally excluded [105]. Moreover, a normalization of PR interval has been documented with ERT [106]. Likewise, in PRKAG2 glycogen-storage cardiomyopathy, the short PR interval was shown to be directly caused by glycogen storage in and around the AV node, being reverted by glycogen depletion in the heart [107]. Still, although characteristic, short PR interval is not common; it has been found in only 14% of FD patients [108]. 

As FD progresses, GB3 accumulation and fibrosis lead to the development of AV and bundle-branch blocks [22,109,110] (Figure 6) and sinus node dysfunction [111], which may require a pacemaker. Bradycardia at rest is common (72%) [112]. Chronotropic incompetence with exercise due to autonomic nervous dysfunction may also occur and contribute to heart failure; hence, exercise stress testing or cardiopulmonary exercise testing could be useful in the differential diagnosis of dyspnea [112,113]. Bradyarrhythmic events were reported in 23% of the patients and were associated with age, LV mass, ejection fraction, and LA strain [114]. There was also a positive correlation between LV mass on cardiac MRI and QRS duration [57]. PR interval duration > 200 ms was reported in 3% and QRS interval duration > 120 ms in 9%. The durations of PR and QRS intervals were shown to increase with age and were identified as independent predictors of need of a pacemaker [111]. In a late-onset phenotype with predominant cardiac involvement, bifascicular block was reported in 25.3% of males and 5.7% of females with a mean age at diagnosis of 62 ± 6 years in males and 78 ± 6 years in females, and complete AV block was reported in 12.7% of males and 1.6% of females with a mean age at diagnosis of 60 ± 7 years in males and 79 ± 6 years in females. The frequency of bundle branch blocks and complete AV block increased with age as well as the duration of QRS interval [4,5].

#### 3.2.2. Tachydysrhythmias

GB3 deposition in the atria [115] and subsequent fibrosis, together with LVH and diastolic dysfunction and atrial dilation, are the proposed mechanisms of development of atrial fibrillation [98], which was reported in 3% of FD patients [111]. Another study reported that 3.9% of the patients had persistent atrial fibrillation and 13.3% had paroxysmal atrial fibrillation, and age was the only independent predictor of this dysrhythmia [98]. Its annual incidence was reported as 1 per 100 person-years and age and LV mass were independent predictors of its development [100]. In a late-onset phenotype with predominant cardiac involvement, atrial fibrillation was found in 7.6% of males and 2.4% of females, while atrial flutter in 2.5% and 0.8%, respectively. Mean age at diagnosis of atrial fibrillation was 67 ± 11 years in males and 77 ± 3 years in females [4,5].

A study of 1448 untreated Fabry patients showed ventricular arrhythmias in 13% of men and 20% of women [116]. In a further study, non-sustained VT was reported in 21%, while sustained VT in only 1% and bradycardia requiring device in 6% of Fabry patients during a median follow-up of 3.6 years, suggesting a higher impact of bradyarrhythmic clinical events [117]. A systematic review of the literature estimated the occurrence of VT in 15.3% of the patients and reported that 75% of the deaths were due to cardiac disease and 62% due to SCD, ranging the incidence of SCD events from 0.34% to 1.4% per annum [67]. In a late-onset phenotype with predominant cardiac involvement, non-sustained VT (Figure 6) was found in 14.1% of males and 5.6% of females, with a mean age at diagnosis of 57 ± 8 and 70 ± 6 years, respectively [4,5].

GB3 accumulation in cardiac conduction system has been reported in FD patients presenting with VT in the absence of LVH, suggesting that GB3 deposits may precipitate VT [118]. However, the major mechanism of sustained VT in FD appears to be re-entry related to myocardial fibrosis [119]. 

Indeed, the annual increase in fibrosis during follow-up was identified in a study as the only independent predictor of malignant ventricular arrhythmias [66]. Other study showed that SCD only occurred in patients with documented VT and LGE [120]. Patients with elevated LV mass index also had more overall arrhythmia, ventricular arrhythmia, and sustained VT [50]. Finally, a systematic review of the literature identified age, male gender, LVH, LGE, and non-sustained VT as risk factors associated with SCD events [67].

Sympathetic nerve damage, demonstrated by MIBG defects in the inferolateral wall, occurs in FD patients without LGE and, in patients with LGE, MIBG defects are larger than the LGE areas, suggesting that sympathetic nerve damage precedes myocardial fibrosis [121]. Therefore, MIBG may have a unique role in assessing the risk of ventricular arrhythmia and SCD [36].

Dysrhythmias occur especially in late stages of the disease [22]. Implantable loop recorders (ILR) revealed clinically relevant dysrhythmias, including four episodes of asystole, seven of bradycardia, five of paroxysmal atrial fibrillation, and five of VT (three sustained and two non-sustained) in 16 patients with advanced cardiomyopathy, with no abnormalities on Holter, followed for a median of 1.2 years. These findings led to new management decisions (pacemaker or ICD implantation, anticoagulation, and termination of beta-blockers) in more than half of them (n = 9) [122].

#### 3.2.3. Cardiac Devices

In a study, the annual rate of cardiac device implantation was estimated at 1.90 per 100 person-years. A pacemaker was needed in 12.5% of the patients due to AV blocks or sinus node dysfunction, and an implantable cardioverter-defibrillator (ICD) was placed in 4.2% due to non-sustained VT. Age at diagnosis of FD and age at the last follow-up visit were independently associated with an increased risk of rhythm/conduction abnormalities requiring cardiac device [123]. The annual incidence of cardiac device implantation for the treatment of bradycardia was 1.07 per 100 person-years and age and QRS duration were independent predictors of device implantation [100]. Another study reported a 5-year cumulative incidence of pacemaker implantation of 8%, which is more than 25 times greater than in general population [111]. In a late-onset phenotype with a predominant cardiac involvement, a pacemaker was implanted in 12.7% of males and 2.4% of females and an ICD in 1.3% and 0.8%, respectively. Mean survival free from pacemaker was 71 ± 2 and 86 ± 1 years, respectively [4,5] (Table 3).

Patients with devices were older, had greater LV mass, more scar tissue and larger atrial size. A class I indication for device implantation was found in 92% of the patients with permanent pacemakers, but in only 28% of the patients with ICD. Moreover, further 44% of patients had ICD inserted for primary prevention outside of current guidance [124].

**Table 3:** Frequency of dysrhythmias and cardiac conduction disorders in FD.

Cardiac Manifestations	Frequencies	References
Palpitations	15.3% in untreated males 21.3% in untreated females	Linhart et al. [34]
Syncope	5.6% in untreated males 2.4% in untreated females	Linhart et al. [34]
Short PR interval	14% of patients	Namdar et al. [108]
Bradycardia	72% of patients	Lobo et al. [112]
Bradyarrhythmic events	23% of patients	Di et al. [114]
PR interval > 200 ms	3% of patients	O’Mahony et al. [111]
QRS duration > 120 ms	9% of patients	O’Mahony et al. [111]
Right bundle branch block	In late-onset phenotype with predominant cardiac involvement 38.8% of males Mean age at diagnosis of 61 ± 6 years 8.5% of females Mean age at diagnosis of 76 ± 9 years	Azevedo et al. [5]
Left anterior fascicular block	In late-onset phenotype with predominant cardiac involvement 45.5% of males Mean age at diagnosis of 60 ± 7 years 10.3% of females Mean age at diagnosis of 72 ± 14 years	Azevedo et al. [5]
Bifascicular block	In late-onset phenotype with predominant cardiac involvement 25.3% of males Mean age at diagnosis of 62 ± 6 years 5.7% of females Mean age at diagnosis of 78 ± 6 years	Azevedo et al. [5]
Complete AV block	In late-onset phenotype with predominant cardiac involvement 12.7% of males Mean age at diagnosis of 60 ± 7 years 1.6% of females Mean age at diagnosis of 79 ± 6 years	Azevedo et al. [5]
Atrial Fibrillation	3% of patients	O’Mahony et al. [111]
	3.9% of patients had persistent atrial fibrillation 13.3% of patients had paroxysmal atrial fibrillation	Shah et al. [98]
	Annual incidence: 1 per 100 person-years	Patel et al. [100]
	In late-onset phenotype with predominant cardiac involvement 7.6% of males Mean age at diagnosis of 67 ± 11 years 2.4% of females Mean age at diagnosis of 77 ± 3 years	Azevedo et al. [5]
Atrial Flutter	In late-onset phenotype with predominant cardiac involvement 2.5% of males 0.8% of females	Azevedo et al. [5]
Ventricular Arrhythmias	13% in males 20% in females	Pinderski et al. [116]
	Non-sustained VT in 8.3% of patients during 1.9 years of follow-up (n = 78)	Shah et al. [98]
	Non-sustained VT in 21% of patients Sustained VT in 1% of patients (Median follow-up of 36 months)	Hanneman et al. [117]
	Systematic review of the literature VT in 15.3% of the patients	Baig et al. [67]
	In late-onset phenotype with predominant cardiac involvement Non-sustained VT in 14.1% of males Mean age at diagnosis of 57 ± 8 years Non-sustained VT in 5.6% of females Mean age at diagnosis of 70 ± 6 years	Azevedo et al. [5]
SCD	SCD in 1 patient during 1.9 years of follow-up (n = 78)	Shah et al. [98]
	VF in 1 patient in the setting of multiorgan failure during mean follow-up of 53 months (n = 19)	Acharya et al. [125]
	Systematic review of the literature 62% of cardiac deaths are sudden Incidence of SCD events: 0.34–1.4% per annum	Baig et al. [67]
Cardiac device	Annual incidence: 1.9 per person-years	Sené et al. [123]
	Annual incidence of devices for the treatment of bradycardia (pacemaker/ICD): 1.07 per person-years	Patel et al. [100]
Pacemaker	12.5% of patients	Sené et al. [123]
	5-year incidence: 8%	O’Mahony [111]
	In late-onset phenotype with predominant cardiac involvement 12.7% in males Mean survival free from pacemaker: 71 ± 2 2.4% in females Mean survival free from pacemaker: 86 ± 1	Azevedo et al. [5]
ICD	4.2% of patients	Sené et al. [123]
	In late-onset phenotype with predominant cardiac involvement 1.3% in males 0.8% in females	Azevedo et al. [5]

AV, atrioventricular; ICD, implantable cardioverter-defibrillator; SCD, sudden cardiac death; VF, ventricular fibrillation; VT, ventricular tachycardia.

### 3.3. Coronary Manifestations

GB3 accumulation results in hypertrophy and proliferation of smooth muscle and endothelial cells and widespread narrowing of intramural coronary arteries [21,126]. 

On angiography, epicardial coronary arteries have been described as structurally normal, but presenting slow flow, and small vessel disease correlated with coronary slow flow and extent of fibrosis [126].

Hence, FD patients with angina present myocardial ischemia on exercise stress test, myocardial perfusion defects on single-photon emission computed tomography [126], and reduced myocardial blood flow and coronary flow reserve on positron emission tomography (PET) [127,128,129]. Myocardial blood flow is reduced in patients without LVH, both males and females, although it is more reduced in patients with LVH [129]. Perfusion defects showed marked regional heterogeneity, with prevalent hypoperfusion of the apical region [129]. In cardiac MRI including vasodilator stress perfusion mapping, the stress myocardial blood flow is also lower in all FD patients regardless of LVH status, although the reduction is more pronounced in patients with LVH. The reduction in stress myocardial blood flow is more pronounced in the subendocardium than in the subepicardium. LGE and low T1 were identified as independent predictors of stress global myocardial blood flow, while LV wall thickness, LGE, and T2 value as predictors of stress segmental myocardial blood flow [130].

Besides coronary microvascular dysfunction, coronary vasospasm may also contribute to angina [131,132] and may precipitate dysrhythmias and sudden death [133].

Oxygen supply to myocardium may also be impaired by LVOT obstruction and increased LV diastolic filling pressure, which reduce blood perfusion of the subendocardial layer of myocardium. Taken together with an increased demand of oxygen by the hypertrophied myocardium, the result is myocardial ischemia, which may be asymptomatic or manifest as angina and myocardial infarct or contribute to dysrhythmias and heart failure [134,135].

Moreover, FD patients commonly present cardiovascular risk factors and may develop coronary atherosclerotic disease. It is not clear if FD may increase the risk of accelerated atherosclerosis [136]. 

Angina has been reported in 22% of males and 23% of females with FD [134]. Mean age of onset was reported to be 42 ± 5 and 49 ± 13 years in males and females, respectively [31]. It may be the first manifestation of FD cardiomyopathy, preceding the development of LVH [137]. However, myocardial infarct is rare (<2%) [134], occurring in 2.7% of males and 1.5% of females [138] (Table 4). Hence, FD remains a rare cause of chest pain in patients without obstructive coronary artery disease (0.15%) [139].

### 3.4. Valvular Disease

GB3 accumulation in valve fibroblasts may ultimately lead to valve thickening and fibrosis [18,19] and regurgitation [20,35], which are usually mild/moderate and rarely require intervention [25,35]. Left-sided valves are most affected, probably due to greater hemodynamic stress [134]. Aortic valve was reported to be affected in 47% of the cases and mitral valve in 57% [35] (Table 4). Mitral valve prolapse has also been described [140], although now known to be less prevalent than originally reported [35]. Aortic root dilation may also occur in late stages of the disease, thereby contributing to aortic regurgitation [35]. The development of valve dysfunction also contributes to heart failure [25].

### 3.5. Aortic Dilation

Degenerative changes in the aortic media from glycolipid deposition [19] can lead to aortic dilatation at the sinuses of Valsalva and ascending aorta in 32.7% and 29.6% of males and 5.6% and 21.1% of females, respectively [141]. An aortic aneurysm, defined as an aortic diameter exceeding 1.5 times the upper limit of normal at the sinuses of Valsalva, was found in 9.6% of male and 1.9% of female patients [141] (Table 4).

**Table 4:** Frequency of other cardiac manifestations in FD.

Cardiac Manifestations	Frequencies	References
Angina	22% in males 23% in females	Linhart et al. [134]
	Mean age of onset in males: 42 ± 5years Mean age of onset in females: 49 ± 13 years	Mehta et al. [31]
Myocardial infarct	<2%	Linhart et al. [134]
	2.7% in males 1.5% in females	Patel et al. [138]
Aortic valve dysfunction	47% of patients	Linhart et al. [35]
Mitral valve dysfunction	57% of patients	Linhart et al. [35]
Aortic dilation	At the sinuses of Valsalva 32.7% in males 5.6% in females At the ascending aorta 29.6% in males 21.1% in females	Barbey et al. [141]
Aortic aneurysm	9.6% in males 1.9% in females	Barbey et al. [141]

### 3.6. Cardiac Events

The incidence of adverse cardiac events (composite endpoint of VT, bradycardia requiring device implantation, severe heart failure, or cardiac death) was 7.6% per year; LVH and LGE were predictors of adverse cardiac events and patients with extensive LGE (≥15% of LV mass) were at highest risk [117]. In another study, the incidence of the primary endpoint (a composite of new onset atrial fibrillation, NYHA ≥ III symptoms, device insertion for bradycardia, or cardiac death) was 2.64 per 100 person-years. Age, MSSI, and QRS duration were independent predictors of the primary endpoint [100]. Age, eGFR, high-sensitivity troponin I, NT-proBNP, LV mass index, E/E’ ratio, and GLS have shown incremental value in the prediction of adverse cardiac events (defined as composite of cardiac death, malignant VT, atrial fibrillation, or severe heart failure) [142] (Table 5).

Cardiac events (defined as myocardial infarction, arrhythmia, angina pectoris, congestive heart failure, or significant cardiac procedures, such as pacemaker placement, coronary bypass, stent placement, and valve replacement) were reported in 69.9% of males and 81.6% of females. Cardiac events were the first clinical events in 21.4% of males and 16.9% of females. Age at first cardiac event was 41.7 (5.3–80.4) in males and 49.8 (17.3–78.2) in females [28].

Ultimately, heart disease is the main cause of death of Fabry patients (40% in males and 41.7% in females) [28]. Cardiac death occurred in 3.4% over a mean follow-up period of 7.1 years (2.4% due to SCD and 1.0% due to heart failure). The annual incidence of cardiac death was 0.52 per 100 person years and the only independent predictor was LV mass [100]. Cardiac death was reported to occur at a median age of 55.5 years in males and 66.0 years in females [28]. All-cause mortality and heart failure-related mortality is higher in patients with FD than with sarcomeric HCM [144].

Table 6 summarizes the main recommendations for the diagnosis and monitoring of cardiac manifestations in FD.

## 4. Late-Onset Phenotypes with Predominant Cardiac Involvement (“Cardiac Variants”)

Cardiac involvement may be the predominant feature in some late-onset phenotypes. These phenotypes, commonly designated by some authors as “cardiac variants” of FD, have been described in association to the *GLA* mutations p.F113L [4,5,146], p.N215S [147,148,149,150], IVS4+919G>A [151], p.A20P [152], p.I91T [146], p.N139S [153], p.I232T [154], p.I239M [155], p.Q279E [156,157], p.M296I [152,158], p.M296V [159], p.R301Q [156,157,160], and p.G328R [161].

Some of these mutations are very common and, despite their widespread distribution around the world, they occur in large clusters in specific geographical areas, such as the IVS4+919G>A mutation in Taiwan, the p.N215S mutation in the United Kingdom, and the p.F113L mutation in the Portuguese region of Guimarães. Indeed, a founder effect has already been documented for the IVS4+919G>A mutation in Southern China [162] and the p.F113L mutation in the Portuguese region of Guimarães [4,29].

These *GLA* mutations are associated to residual enzymatic activity of α-galactosidase A [4,5,150,163]. Plasma lyso-GB3 levels are lower than in patients with classic phenotypes in both genders, and normal or near-normal values are usually found in these females [4,5,150,164,165,166,167,168,169]. Cardiac deposits of GB3 are only found in myocardial cells [22,164,167,170,171].

Cardiac manifestations are common and carry the highest prognostic impact [4,5]. Despite the attenuated course of late-onset phenotypes, the severity of cardiac involvement is the same or greater than in classic phenotypes [164,172]. LVH remains the most common cardiac manifestation, occurring in 40.2% of the patients (21–67%) [5]. The first cardiac manifestations are LVH and LGE, which arise in males over 30 years and are followed by heart failure, non-sustained VT, and cardiac conduction disorders, which arise in males over 40 years, culminating with the development of bifascicular block and complete AV block in males beyond the age of 50 years. Cardiac manifestations are more common and arise one to two decades earlier in males, and their frequency and severity increase with age in both genders [5].

In these late-onset phenotypes, acroparesthesias, neuropathic pain, hypohidrosis, heat, cold and exercise intolerance, gastrointestinal symptoms, angiokeratomas, and cornea *verticillata* are characteristically absent or rare [4,5,150,163,165,166,169,172]. However, other extracardiac manifestations, such as proteinuria, brain white matter lesions, and deafness, are common and arise early, before 30 years of age. Their prognostic impact, however, is lower, as renal insufficiency, stroke, and need for a hearing device is uncommon [4,5,150,165,166,169,172,173,174].

## 5. Cardiac Treatment in FD

### 5.1. ERT

ERT with recombinant α-galactosidase A has been approved for clinical use since 2001. There are two commercially available preparations, agalsidase alfa and agalsidase beta, both administered intravenously every other week at the doses of 0.2 and 1 mg/kg of body weight, respectively [175].

According to the current recommendations, ERT should be initiated in classic males at the age of 16 years regardless of symptomatic status, although it should be considered earlier, on an individual basis, since the age of 8–10 years old. In late-onset males and in classic/late-onset females, LVH, cardiac fibrosis or cardiac rhythm, or conduction abnormalities constitute indications to start ERT [176].

Agalsidase alfa has been demonstrated to decrease/stabilize LV mass [177,178,179] and wall thickness [179] in males, as well as to decrease/stabilize LV mass [178,179,180,181] and wall thickness [179] and to improve exercise capacity [181] in females. However, myocardial GB3 content did not significantly decrease on endomyocardial biopsies taken at 6 months of treatment with agalsidase alfa [177]. In a study comparing patients treated with agalsidase alfa from the Fabry Outcome Survey registry with historical cohorts of untreated patients, the median age at first event seemed to be higher in patients under agalsidase alfa than in untreated patients, both in males (48 vs. 41 years) and females (57 vs. 53 years). The median survival time also seemed to be higher in males under agalsidase alfa than in untreated males (77.5 vs. 60 years). However, these findings have to be interpreted with caution because this study was a retrospective analysis that used a historical cohort as a comparator group [182].

Agalsidase beta has been shown to reduce/stabilize LV mass [128,183,184,185] and wall thickness [128,185] in males, as well as to decrease/stabilize LV mass [183,185] and wall thickness [185] in females. Complete clearance of GB3 deposits was also achieved in interstitial capillary endothelial cells from 72% of the patients treated with agalsidase beta for 5 months, and this benefit seemed to be sustained up to 60 months. Nevertheless, no clearance of GB3 was observed in the cardiomyocytes [186]. Conversely, in another study, there was no improvement of GB3 deposits in endothelial and smooth muscle cells, myocardial blood flow, perfusion defects, or electrocardiographic changes on exercise stress test with agalsidase beta for ≥12 months [126]. In a randomized clinical trial, agalsidase beta, compared to placebo, significantly increased the time to first clinical event in protocol-adherent patients, when adjusted for baseline proteinuria (considering clinical events as cardiac: myocardial infarction; new symptomatic arrhythmia requiring antiarrhythmic medication, pacemaker, direct current cardioversion, or defibrillator implantation; unstable angina defined by national practice guidelines and accompanied by electrocardiographic changes resulting in hospitalization; or worsening congestive heart failure requiring hospitalization; renal: 33% increase in serum creatinine level from baseline (2 consecutive values) or end-stage kidney disease requiring long-term dialysis or transplantation; cerebrovascular: stroke or transient ischemic attack; or death). However, most clinical events were renal and these were limited to the increase in serum creatinine, while there was a low rate of cerebrovascular events and death [187]. Moreover, agalsidase beta has reduced the incidence rate of clinicals events after 6 months of treatment, maintaining, from 6 months to up to 5 years of treatment, a stable incidence rate of clinical events (cardiac: myocardial infarction, first-time congestive heart failure, atrial fibrillation, VT, evidence of progressive heart disease sufficiently severe to require a pacemaker, heart bypass surgery, coronary artery dilatation or implantation of an ICD; renal: chronic dialysis (>40 days) or renal transplantation; cerebrovascular: hemorrhagic or ischemic stroke; or death due to any cause) [188].

The evaluation of ERT efficacy in the late-onset phenotypes with predominant cardiac involvement has been limited to a few small studies in the IVS4+919G>A mutation. In these patients, a significant negative correlation has been described between ERT duration and GB3 accumulation in cardiomyocytes and cardiomyocyte size [189]. In patients under ERT for more than 3 years, GB3 deposits were not found in cardiomyocytes [170]. In a study including 23 patients with the IVS4+919G>A mutation, plasma lyso-Gb3 decreased under ERT both in males and females, reaching the lowest value at 11.1 months and increasing gradually thereafter, even when LV mass index was still improving or remaining stable [167]. Finally, in another study, including 26 patients with the IVS4+919G>A mutation under ERT for 6–39 months, a significant reduction in mean plasma lyso-GB3 (on average by 28%) has been reported in males and females, with 89% of patients experiencing a reduction in plasma lysoGB3. A stabilization or reduction in LV mass index, interventricular septum and posterior wall thickness has been observed in 83%, 83%, and 67% of patients, respectively, with an average decrease in 12% in LV mass index, 14% in interventricular septum, and 13% in posterior wall thickness. Mean LV mass index, interventricular septum, and posterior wall thickness have been found to decrease both in males and females, although only reaching statistical significance for LV mass index and posterior wall thickness in females [166].

Better cardiac outcomes are achieved with early treatment. Agalsidase beta was associated to a statistically significant decline in LV mass when started at the age < 30 years, but an increase in LV mass was seen when it was started at the age ≥ 50 years [184]. Likewise, agalsidase beta started at the age < 40 years was associated to a stable thickness of the interventricular septum and posterior wall over a period of 10 years, whereas a significant worsening of these parameters was observed over time when it was started at the age ≥ 40 years [190]. Moreover, in patients without fibrosis, agalsidase beta resulted in a statistically significant decline of LV mass and improvement of exercise capacity and LV radial strain rate, while no effect was observed in patients with mild or severe fibrosis at the time of treatment initiation [65]. Similarly, in patients treated with agalsidase alfa, LVH or low eGFR at the time of treatment initiation were associated with a higher risk of cardiovascular events [191]. 

A study comparing agalsidase alfa and agalsidase beta found that a higher proportion of patients had a decrease in LV mass index when treated for 1 year with agalsidase beta than with agalsidase alfa at licensed doses (79% vs. 62%). Nevertheless, no difference between agalsidase alfa and beta was found regarding clinical events [192].

Neutralizing anti-drug antibodies may attenuate ERT efficacy. Although previous reports have suggested that anti-drug antibodies had no effect on the time to first clinical event [193], other studies have shown that they were associated to higher LV mass, disease severity scores, and frequency of symptoms [194] and worse renal function [194,195].

### 5.2. Migalastat

Migalastat is a first-in-class pharmacological chaperone therapy for FD, administered orally at the dosage of 123 mg once every other day, which has been approved by the European Medicines Agency for the treatment of FD patients aged ≥ 16 years, with eGFR ≥ 30 mL/min/1.73 m^2^ and amenable *GLA* mutations [175,196].

Migalastat has consistently shown to decrease LV mass [197,198,199,200,201,202,203]. In the FACETS trial, in the modified-intention to treat population (i.e., ERT-naïve patients with migalastat-amenable *GLA* mutations), there was a significant reduction in the mean LV mass index compared to the baseline after 24 months of migalastat therapy (i.e., after 18 months of migalastat in patients who switched from placebo or 24 months of continuous migalastat) [197]. In the ATTRACT trial, in ERT-experienced patients with amenable *GLA* mutations who were randomized to switch to migalastat or continue ERT, migalastat significantly reduced the mean LV mass index at 18 months, and changes on LV mass index correlated with changes in the thickness of the interventricular septum and not the posterior wall [198]. In the open-label extension study, a significant decrease in LV mass index was found after 30 months of migalastat in patients with LVH at baseline [199]. Muntze et al. reported one patient who showed improvement of LV mass, LGE, troponin, and NT-proBNP under treatment with migalastat for 12 months [200]. Later, the same authors reported a decrease in LV mass index in 14 patients treated with migalastat for 1 year [201]. Riccio et al. also reported a significant decrease in LV mass index after 1 year of treatment with migalastat in seven FD males previously treated with ERT [202]. In the larger FAMOUS study, including 59 previously ERT-treated and untreated FD patients, treatment with migalastat for 12 months was also associated to a significant decrease in LV mass index [203].

No data have been published on the efficacy of migalastat specifically on the late-onset phenotypes.

### 5.3. Supportive Treatment

Control of cardiovascular risk factors, including arterial hypertension and dyslipidaemia is indicated [134]. Systolic blood pressure was higher in patients with LGE and highest in patients with a faster progression of LGE [204]. In fact, hypertension increased the odds of cardiovascular events (myocardial infarction, heart failure, or cardiac-related death) by 7.8 in men and 4.5 in women [138].

Angiotensin converting enzyme inhibitors or angiotensin II receptor blockers should be used in patients with LVH, LV systolic dysfunction, and heart failure, but also in the presence of proteinuria [25,51,145,205,206]. Mineralocorticoid receptor antagonists should also be considered in patients with heart failure and LV systolic dysfunction [51,145]. However, these drugs must be used with caution in patients with nephropathy due to the possible development of hyperkalemia or worsening of renal function [145]. Evidence is lacking on the use of sacubitril/valsartan in Fabry patients. 

Beta-blockers are recommended to relieve LVOT obstruction symptoms or control the rate of atrial fibrillation/flutter and should be considered in patients with angina or heart failure and LV systolic dysfunction [25,51,145,207,208]. Verapamil should also be considered for the treatment of angina and is recommended for the treatment of LVOT obstruction symptoms. Diltiazem should also be considered in patients with LVOT obstruction symptoms or angina. Ivabradine should be considered for the treatment of heart failure or angina, according to the European Society of Cardiology (ESC) guidelines [51,145,207,208]. However, these drugs must be handled with care, due to the propensity of FD patients to develop chronotropic incompetence and bradydysrhythmias. Dihydropyridine calcium channel blockers may be safer alternatives and should be considered for angina treatment [134,208]. 

Loop diuretics should be considered to treat symptoms of congestion in patients with heart failure [134,207]. 

Cardiac resynchronization therapy (CRT) has also been applied in a few FD cases at burn-out stage with LV systolic dysfunction and heart failure [209], but its efficacy in this cohort of patients may be hampered by extensive scar burden. Nevertheless, it should be considered in patients with LV dysfunction (ejection fraction ≤ 35%), according to the current ESC guidelines [207]. CRT-P should also be considered in symptomatic patients with a pacing indication, LV ejection fraction < 50%, and QRS duration > 120 ms [145].

In patients with resting or latent LVOT obstruction, digoxin is not recommended and vasodilators, such as angiotensin converting enzyme inhibitors, angiotensin II receptor blockers, dihydropyridines, and nitrates, should be avoided, if possible [51,145]. Myectomy should be performed in the rare cases of LVOT obstruction associated with severe symptoms of heart failure or syncope, resulting in excellent operative outcome and relief of LVOT obstruction and symptoms [210]. Septal alcohol ablation may be a safe alternative treatment for alleviation of LVOT obstruction and improvement of related heart failure in Fabry patients [211].

Antiplatelet aggregation therapy should be started in patients who suffered a stroke or myocardial infarction [134]. Anticoagulation should be immediately started once atrial fibrillation or flutter is detected [134], based on the evidence extrapolated from HCM, and no risk score should be used for this purpose as none has been validated in FD [145]. Direct oral anticoagulants (DOACs) should be considered as the first-line choice in Fabry patients without contraindications, namely, related to renal failure, because, despite the lack of systematic data on their use on FD, they are associated to lower risk of intracranial bleeding and may avoid the risk of warfarin-induced nephropathy [145].

Prior stroke/transient ischemic attack (TIA), angiokeratoma, LV posterior wall thickness >14 mm, creatinine ≥1.0 mg/dL, and GLS > −13.5% were identified as independent risk factors for new or recurrent stroke/TIA in FD patients without atrial fibrillation. A new predicting score based on these risk factors was proposed to predict stroke/TIA in FD patients without atrial fibrillation. It remains to clarify if high-risk patients, according to this score, might benefit from antithrombotic therapy [212]. 

Amiodarone should be avoided in FD patients, as it induces lysosomal dysfunction and may precipitate clinical worsening [213]. It induces phospholipidosis by inhibiting lysosomal degradation of phospholipids [214]. Additionally, dronedarone is contraindicated in patients with heart failure (NYHA class III–IV) and renal failure (eGFR < 30 mL/min). Sotalol, flecainide, and propafenone are also contraindicated in patients with heart failure [145].

Isolation of pulmonary veins has been performed [215], but it may require longer and repeated procedures [216].

Pacemaker may be required to treat symptomatic bradycardia or symptomatic/advanced cardiac blocks, according to ESC guidelines [134,145].

ICD implantation is recommended in patients who suffered sudden cardiac arrest due to VT/ventricular fibrillation or sustained VT causing syncope or hemodynamic compromise and have a life expectancy of >1 year [145]. ICD implantation should be considered in patients with advanced hypertrophy and fibrosis, who require pacemaker implantation and have a life expectancy of >1 year [145]. Evidence is lacking to guide ICD implantation in primary prevention in FD patients and the HCM RISK-SCD score should not be used for this purpose [145]. Nevertheless, it is generally accepted that FD patients should receive ICD in the presence of heart failure (NYHA class II–III) and LV ejection fraction of ≤35% [207]. Patients who have significant fibrosis on MRI and those who have non-sustained VT on Holter monitoring are at higher risk for arrhythmic complications and may be considered for ICD [66,123,145]. VT ablation has also been performed with success in some cases of recurrent VT or ICD storms [119]. However, radiofrequency ablation by endocardial and/or epicardial approaches is challenging, as the target tissue is frequently localized at the midwall [216]. Asymptomatic runs of non-sustained VT do not usually require anti-arrhythmic therapy [145].

Heart transplantation should be considered in patients with advanced heart failure with severe LV systolic and diastolic dysfunction and NYHA class III–IV despite optimal medical therapy, or intractable ventricular arrhythmia, depending on the extension of the extracardiac involvement by the disease and considering that the disease does not affect the transplanted organ [134,217] (Table 7).

## 6. Conclusions

Cardiac involvement remains the leading cause of death in Fabry patients. GB3 deposits are found in virtually all cardiac cells, but the mechanisms leading to organ damage remain less defined. Fabry hearts exhibit myocardial hypertrophy, inflammation, apoptosis, necrosis and fibrosis, valve thickening, and narrowing of intramural coronary arteries. Cardiac manifestations therefore include LVH, heart failure, angina, valve disease, dysrhythmias, cardiac conduction blocks, and sudden cardiac death, and the severity of cardiac involvement is the same either in classic or late-onset phenotypes.

ERT has demonstrated to reduce or stabilize LV mass and wall thickness and to reduce the incidence and delay the occurrence of clinical events, whereas migalastat has consistently shown to reduce LV mass. Early treatment has shown to achieve better outcomes, while cardiac fibrosis is deemed to be irreversible. Still, evidence is lacking on the efficacy of available therapies on hard-endpoints, such as total mortality, cardiovascular mortality, heart failure, dysrhythmias, or need for a cardiac device, as well as in late-onset phenotypes with predominant cardiac involvement. Furthermore, despite several therapies targeting, the enzymatic defect or substrate accumulation are under research, it remains to be clarified if other therapeutic strategies will be needed to avoid or revert cardiac damage in FD.

## Figures and Tables

**Table 1:** Frequency of cardiac symptoms in FD.

Cardiac Manifestations	Frequencies	References
Cardiac signs or symptoms	60% in males Mean age of onset 29.2 ± 14.4 years 50% in females Mean age of onset 34.5 ± 17.6 years	Mehta et al. [26]
Cardiac symptoms as presenting symptoms	13% in males 10% in females	Eng et al. [27]

**Table 2:** Frequency of manifestations related to ventricular hypertrophy, fibrosis and dysfunction in FD.

Cardiac Manifestations	Frequencies	References
LVH	43% in males Mean age of onset 39 ± 10 years 26% in females Mean age of onset 50 ± 11 years	Mehta et al. [31]
	76.9% in patients ≥ 75 years	Lidove et al. [33]
	In late-onset phenotype with predominant cardiac involvement 73.1% of males Mean age at diagnosis of 57 ± 10 years 19.0% of females Mean age at diagnosis of 73 ± 8 years	Azevedo et al. [5]
LGE	50% of patients	Moon et al. [48]
	In late-onset phenotype with predominant cardiac involvement 21.4% of patients	Azevedo et al. [5]
LV diastolic dysfunction	63% of patients with LGE	Niemann et al. [77]
	In late-onset phenotype with predominant cardiac involvement 29.2% of patients 69.4% of patients with LVH	Azevedo et al. [4]
LV systolic dysfunction (reduced EF)	6.7% of patients	Wu et al. [80]
Latent LVOT obstruction	43% of patients	Calcagnino et al. [90]
RV hypertrophy	31–71% of patients	Niemann et al. [92] Graziani et al. [93]
Heart Failure (or Dyspnoea)	19.4% in untreated males 19.7% in untreated females	Linhart et al. [34]
	34.6% in patients ≥ 75 years	Lidove et al. [33]
	Severe heart Failure (NYHA class ≥III) in 10% Annual incidence of severe heart failure: 1.62 per 100 person-years	Patel et al. [100]
	In late-onset phenotype with predominant cardiac involvement 32.9% of males Mean survival free from heart failure: 64 ± 1 years 14.8% of females Mean survival free from heart failure: 76 ± 2 years	Azevedo et al. [5]

EF, ejection fraction; LGE, late gadolinium enhancement; LV, left ventricular; LVH, left ventricular hypertrophy; LVOT, left ventricular outflow tract; NYHA, New York Heart Association; RV, right ventricular.

**Table 5:** Predictors/Factors associated to cardiac events in FD.

Cardiac Endpoints	Predictors/Factors Associated to Outcome	References
Composite endpoint of VT, bradycardia requiring device implantation, severe heart failure or cardiac death	Predictors LVH LGE Extensive LGE (≥15% of LV mass) were at highest risk	Hanneman et al. [117]
Composite endpoint of new onset atrial fibrillation, NYHA ≥ III symptoms, device insertion for bradycardia or cardiac death	Predictors Age MSSI QRS duration	Patel et al. [100]
Composite endpoint of myocardial infarction, heart failure, or cardiac-related death	Predictor LVH	Patel et al. [138]
Composite endpoint of death, myocardial infarction, cerebral vascular accident, exacerbation of heart failure, arrythmia, or implantation of permanent pacemaker or cardioverter-defibrillator	Associated factor Impaired basal segmental longitudinal strain	Zada et al. [84]
Composite endpoint of sudden death, arrhythmia or pacing device insertion	Predictor End-stage renal disease	Talbot et al. [143]
Composite of cardiac death, malignant VT, atrial fibrillation or severe heart failure	Predictors Age eGFR High-sensitivity troponin I NT-proBNP LV mass index E/E’ ratio GLS	Spinelli et al. [142]

eGFR, estimated glomerular filtration rate; GLS, global longitudinal strain; LGE, late gadolinium enhancement; LV, left ventricular; LVH, left ventricular hypertrophy; MSSI, Mainz severity score index; NT-proBNP, N-terminal prohormone of brain natriuretic peptide; NYHA, New York Heart Association; VT, ventricular tachycardia.

**Table 6:** Summary of the main recommendations for the diagnosis and monitoring of cardiac manifestations in FD [145].

**Recommendations for the Diagnosis and Monitoring of Cardiac Manifestations in FD**
ECG
A standard 12-lead ECG is recommended in all adult patients at first clinical evaluation, every 6–12 months and when there is development of new symptoms.
**Echocardiogram**
Echocardiogram is recommended in all patients at baseline, every 12–24 months and with the development of new symptoms.
**Exercise echocardiography**
Exercise echocardiography should be performed in all symptomatic patients to exclude latent obstruction and exercise-induced mitral regurgitation.
**Cardiac MRI**
Cardiac MRI should be considered in all adult patients at baseline to assess cardiac morphology and function and myocardial fibrosis; and may be considered, every 2–5 years in patients without cardiac abnormalities and every 2–3 years in patients with progressive disease, in order to assess progression of fibrosis and cardiac function. T1 mapping may also be considered to detect early cardiac involvement or to help in the differential diagnosis of LVH.
**Holter monitoring**
A 24 h-Holter monitoring should be considered in all adult patients at first clinical evaluation, every 6–12 months and when there is development of new symptoms.
**ILR**
A prolonged Holter monitoring or preferably an ILR should be considered in patients with recurrent episodes of unexplained syncope. An ILR may also be considered in patients with palpitations or recent stroke and negative Holter monitoring.
**Cardiopulmonary exercise testing**
Cardiopulmonary exercise testing should be considered in patients with exercise intolerance.
**Coronary angiography**
Coronary angiography (or CT coronary angiography) is recommended in all patients with angina CCS class ≥ II. Invasive coronary angiography is recommended in adult survivors of cardiac arrest, in patients with sustained VT and in patients with severe stable angina (CCS class III) or unstable angina.
**BNP/NT-proBNP**
Measurement of plasma BNP/NT-proBNP is recommended in symptomatic patients with suspected heart failure.
**High-sensitivity troponin**
High-sensitivity troponin may be considered to assess disease severity.
**Renal function**
Regular assessment of renal function and albuminuria/proteinuria is recommended in all patients.
**Endomyocardial biopsy**
When a genetic variant of uncertain significance is found in the *GLA* gene, an endomyocardial biopsy with electron microscopy should be considered, particularly in females or in patients with high residual enzyme activity (>10%) and low lyso-GB3 levels, in order to exclude FD as the cause of LVH.

BNP, brain natriuretic peptide; CCS, Canadian Cardiovascular Society; CT, computed tomography; ECG, electrocardiogram; FD, Fabry disease; ILR, implantable loop recorder; LVH, left ventricular hypertrophy; MRI, magnetic resonance imaging; NT-proBNP, N-terminal prohormone of brain natriuretic peptide; VT, ventricular tachycardia.

**Table 7:** Summary of the main recommendations for the supportive treatment of cardiac manifestations in FD.

Recommendations for the Supportive Treatment of Cardiac Manifestations in FD
**Angiotensin Converting Enzyme Inhibitors or Angiotensin II Receptor Blockers ^1,2^**
Angiotensin converting enzyme inhibitors (or angiotensin II receptor blockers, if not tolerated) should be used in patients with LV systolic dysfunction and heart failure [25,51,145].
**Beta-blockers ^3^**
Beta-blockers should be considered in patients with heart failure and LV systolic dysfunction; or in patients with angina [25,51,145,207,208]. Beta-blockers are recommended to relieve LVOT obstruction symptoms and to control the rate of atrial fibrillation/flutter [25,51,145].
**Mineralocorticoid receptor antagonists ^1^**
Mineralocorticoid receptor antagonists should be considered in patients with heart failure and LV systolic dysfunction [51,145].
**Loop diuretics**
Loop diuretics should be considered to treat symptoms of congestion in patients with heart failure [134,207].
**Calcium channel blockers**
Dihydropyridines^2^ should be considered for the treatment of angina [134,208]. Verapamil^3^ is recommended in patients with LVOT obstruction symptoms and should be considered in patients with angina [51,145,208]. Diltiazem^3^ should be considered in patients with LVOT obstruction symptoms or angina [51,145,208].
**Ivabradine ^3^**
Ivabradine should be considered for the treatment of heart failure or angina, according to ESC guidelines [145,207,208].
**Antiplatelet therapy**
Antiplatelet therapy should be started in patients who suffered a stroke or myocardial infarction [134].
**Anticoagulation**
Anticoagulation should be immediately started once atrial fibrillation or flutter is detected [145]. Direct oral anticoagulants (DOACs) should be considered as the first-line choice in patients without contra-indications [145].
**Anti-arrhythmic drugs**
Amiodarone should be avoided in FD [213,214]. Dronedarone is contra-indicated in patients with heart failure (NYHA class III–IV) and renal failure (eGFR < 30mL/min) [145]. Sotalol, flecainide and propafenone are contra-indicated in patients with heart failure [145].
**Management of cardiovascular risk factors**
Control of cardiovascular risk factors, including arterial hypertension, diabetes and dyslipidaemia, is indicated [134].
**Pacemaker**
Pacemaker may be required to treat symptomatic bradycardia or symptomatic/advanced cardiac blocks, according to ESC guidelines [134,145]. Dual chamber pacemakers should be implanted unless patients are in permanent atrial fibrillation [145].
**ICD**
ICD implantation is recommended in patients who suffered sudden cardiac arrest due to VT/fibrillation or sustained VT causing syncope/haemodynamic compromise and have a life expectancy >1 year [209]. ICD implantation should be considered in patients with advanced hypertrophy and fibrosis, who require pacemaker implantation and have a life expectancy >1 year [145]. ICD implantation may be considered in patients with severe LVH and advanced fibrosis or non-sustained VT, who have a life expectancy >1 year [145]. ICD implantation is recommended in patients with heart failure (NYHA class II-III) and LV ejection fraction ≤35%, despite ≥3 months of optimal treatment, who have a life expectancy >1 year [207].
**CRT**
CRT should be considered in patients with LV ejection fraction ≤35%, according to ESC guidelines [207]. CRT-P should be considered in symptomatic patients with a pacing indication, LV ejection fraction <50% and QRS duration >120ms [145].
**Septal reduction therapy (myectomy/alcohol ablation therapy)**
Septal reduction therapy is recommended in patients with a resting or provoked LVOT gradient ≥50 mm Hg, who are in NYHA class III–IV, despite maximum tolerated medical therapy [51,210,211]. Septal reduction therapy should be considered in patients with a resting or provoked LVOT gradient ≥50 mm Hg, who suffer recurrent exertional syncope, despite maximum tolerated medical therapy [51,210,211].
**Heart transplantation**
Heart transplantation should be considered in patients with advanced heart failure with severe LV dysfunction and NYHA class III–IV despite optimal medical therapy, or intractable ventricular arrhythmia, depending on the extension of the extracardiac involvement by the disease [134,217].

CRT, Cardiac resynchronization therapy; eGFR, estimated glomerular filtration rate; ESC, European Society of Cardiology; FD, Fabry disease; ICD, Implantable cardioverter-defibrillator; LV, left ventricular; LVH, left ventricular hypertrophy; LVOT, left ventricular outflow tract; NYHA, New York Heart Association; VT, ventricular tachycardia ^1^ Caution should be taken in Fabry patients with nephropathy due to the risk of hyperkalaemia or worsening of renal function; ^2^ Should be avoided, if possible, in patients with resting/latent LVOT obstruction; ^3^ Caution should be taken due to the increased risk of bradycardia in Fabry patients.

## References

[1] Desnick R.J., Ioannou Y.A., Eng C.M., Scriver C.R., Beaudet A.L., Sly W.S., Valle D., Childs B., Kinzler K.W., Vogelstein B. (2001). Alpha-galactosidase A deficiency: Fabry disease. The Metabolic and Molecular Bases of Inherited Disease.

[2] Germain D.P. (2010). Fabry disease. Orphanet J. Rare Dis..

[3] Arends M., Wanner C., Hughes D., Mehta A., Oder D., Watkinson O.T., Elliott P.M., Linthorst G.E., Wijburg F.A., Biegstraaten M. (2017). Characterization of Classical and Nonclassical Fabry Disease: A Multicenter Study. J. Am. Soc. Nephrol..

[4] Azevedo O., Gal A., Faria R., Gaspar P., Miltenberger-Miltenyi G., Gago M.F., Dias F., Martins A., Rodrigues J., Reimão P. (2020). Founder effect of Fabry disease due to p.F113L mutation: Clinical profile of a late-onset phenotype. Mol. Genet. Metab..

[5] Azevedo O., Gago M.F., Miltenberger-Miltenyi G., Robles A.R., Costa M.A., Pereira O., Vide A.T., Branco G.C., Simões S., Guimarães M.J. (2020). Natural history of the late-onset phenotype of Fabry disease due to the p.F113L mutation. Mol. Genet. Metab. Rep..

[6] Deegan P.B., Baehner A.F., Romero M.-Á.B., A Hughes D., Kampmann C., Beck M. (2005). Natural history of Fabry disease in females in the Fabry Outcome Survey. J. Med. Genet..

[7] Whybra C., Kampmann C., Willers I., Davies J., Winchester B., Kriegsmann J., Brühl K., Gal A., Bunge S., Beck M. (2001). Anderson-Fabry disease: Clinical manifestations of disease in female heterozygotes. J. Inherit. Metab. Dis..

[8] Elleder M., Elstein D., Altarescu G., Beck M. (2010). Subcellular, cellular, and organ pathology of Fabry disease. Fabry Disease.

[9] Miller J.J., Kanack A.J., Dahms N.M. (2020). Progress in the understanding and treatment of Fabry disease. Biochim. Biophys. Acta Gen. Subj..

[10] Lücke T., Höppner W., Schmidt E., Illsinger S., Das A.M. (2004). Fabry disease: Reduced activities of respiratory chain enzymes with decreased levels of energy-rich phosphates in fibroblasts. Mol. Genet. Metab..

[11] Shen J.-S., Meng X.-L., Moore D.F., Quirk J.M., Shayman J.A., Schiffmann R., Kaneski C.R. (2008). Globotriaosylceramide induces oxidative stress and up-regulates cell adhesion molecule expression in Fabry disease endothelial cells. Mol. Genet. Metab..

[12] De Francesco P.N., Mucci J.M., Ceci R., Fossati C.A., Rozenfeld P.A. (2013). Fabry disease peripheral blood immune cells release inflammatory cytokines: Role of globotriaosylceramide. Mol. Genet. Metab..

[13] De Francesco P.N., Mucci J.M., Ceci R., Fossati C.A., Rozenfeld P.A. (2011). Higher apoptotic state in Fabry disease peripheral blood mononuclear cells: Effect of globotriaosylceramide. Mol. Genet. Metab..

[14] Squillaro T., Antonucci I., Alessio N., Esposito A., Cipollaro M., Melone M.A.B., Peluso G., Stuppia L., Galderisi U. (2017). Impact of lysosomal storage disorders on biology of mesenchymal stem cells: Evidences from in vitro silencing of glucocerebrosidase (GBA) and alpha-galactosidase A (GLA) enzymes. J. Cell. Physiol..

[15] Choi S., Kim J.A., Na H.-Y., Cho S.-E., Park S., Jung S.-C., Suh S.H. (2014). Globotriaosylceramide Induces Lysosomal Degradation of Endothelial K Ca 3.1 in Fabry Disease. Arter. Thromb. Vasc. Biol..

[16] Ferraz M.J., Marques A.R.A., Appelman M.D., Verhoek M., Strijland A., Mirzaian M., Scheij S., Ouairy C.M., Lahav D., Wisse P. (2016). Lysosomal glycosphingolipid catabolism by acid ceramidase: Formation of glycosphingoid bases during deficiency of glycosidases. FEBS Lett..

[17] Aerts J.M., Groener J.E., Kuiper S., Donker-Koopman W.E., Strijland A., Ottenhoff R., Van Roomen C., Mirzaian M., Wijburg F.A., Linthorst G.E. (2008). Elevated globotriaosylsphingosine is a hallmark of Fabry disease. Proc. Natl. Acad. Sci. USA.

[18] Ferrans V.J., Hibbs R.G., Burda C.D. (1969). The heart in Fabry’s disease. Am. J. Cardiol..

[19] Desnick R.J., Blieden L.C., Sharp H.L., Hofschire P.J., Moller J.H. (1976). Cardiac valvular anomalies in Fabry disease. Clinical, morphologic, and biochemical studies. Circulation.

[20] Becker A.E., Schoorl R., Balk A.G., van der Heide R. (1975). Cardiac manifestations of Fabry’s disease: Report of a case with mitral insufficiency and electrocardiographic evidence of myocardial infarction. Am. J. Cardiol..

[21] Sheppard M.N., Cane P., Florio R., Kavantzas N., Close L., Shah J., Lee P., Elliott P. (2010). A detailed pathologic examination of heart tissue from three older patients with Anderson–Fabry disease on enzyme replacement therapy. Cardiovasc. Pathol..

[22] Takenaka T., Teraguchi H., Yoshida A., Taguchi S., Ninomiya K., Umekita Y., Yoshida H., Horinouchi M., Tabata K., Yonezawa S. (2008). Terminal stage cardiac findings in patients with cardiac Fabry disease: An electrocardiographic, echocardiographic, and autopsy study. J. Cardiol..

[23] Frustaci A., Chimenti C., Doheny D., Desnick R.J. (2017). Evolution of cardiac pathology in classic Fabry disease: Progressive cardiomyocyte enlargement leads to increased cell death and fibrosis, and correlates with severity of ventricular hypertrophy. Int. J. Cardiol..

[24] Frustaci A., Verardo R., Grande C., Galea N., Piselli P., Carbone I., Alfarano M., Russo M.A., Chimenti C. (2018). Immune-Mediated Myocarditis in Fabry Disease Cardiomyopathy. J. Am. Heart Assoc..

[25] Linhart A., Elliott P.M. (2007). The heart in Anderson-Fabry disease and other lysosomal storage disorders. Heart.

[26] Mehta A., Clarke J.T.R., Giugliani R., Elliott P., Linhart A., Beck M., Sunder-Plassman G., on behalf of the FOS Investigators (2009). Natural course of Fabry disease: Changing pattern of causes of death in FOS—Fabry Outcome Survey. J. Med. Genet..

[27] Eng C.M., Fletcher J., Wilcox W.R., Waldek S., Scott C.R., Sillence D.O., Breunig F., Charrow J., Germain D.P., Nicholls K. (2007). Fabry disease: Baseline medical characteristics of a cohort of 1765 males and females in the Fabry Registry. J. Inherit. Metab. Dis..

[28] Waldek S., Patel M.R., Banikazemi M., Lemay R., Lee P. (2009). Life expectancy and cause of death in males and females with Fabry disease: Findings from the Fabry Registry. Genet. Med..

[29] Azevedo O., Marques N., Reis L., Cruz I., Craveiro N., Antunes H., Lourenço C., Gomes R., Guerreiro R.A., Faria R. (2020). Predictors of Fabry disease in patients with hypertrophic cardiomyopathy: How to guide the diagnostic strategy?. Am. Heart J..

[30] Doheny D., Srinivasan R., Pagant S., Chen B., Yasuda M., Desnick R.J. (2018). Fabry Disease: Prevalence of affected males and heterozygotes with pathogenic GLA mutations identified by screening renal, cardiac and stroke clinics, 1995–2017. J. Med. Genet..

[31] Mehta A., Widmer U., Mehta A., Beck M., Sunder-Plassmann G. (2006). Natural history of Fabry disease. Fabry Disease: Perspectives from 5 Years of FOS.

[32] Kampmann C., Linhart A., Baehner F., Palecek T., Wiethoff C.M., Miebach E., Whybra C., Gal A., Bultas J., Beck M. (2008). Onset and progression of the Anderson–Fabry disease related cardiomyopathy. Int. J. Cardiol..

[33] Lidove O., Barbey F., Niu D.-M., Brand E., Nicholls K., Bizjajeva S., Hughes D.A. (2016). Fabry in the older patient: Clinical consequences and possibilities for treatment. Mol. Genet. Metab..

[34] Linhart A., Kampmann C., Zamorano J.L., Sunder-Plassmann G., Beck M., Mehta A., Elliott P.M., on behalf of European FOS Investigators (2007). Cardiac manifestations of Anderson-Fabry disease: Results from the international Fabry outcome survey. Eur. Heart J..

[35] Linhart A., Paleček T., Bultas J., Ferguson J.J., Hrudová J., Karetová D., Zeman J., Ledvinová J., Poupětová H., Elleder M. (2000). New insights in cardiac structural changes in patients with Fabry’s disease. Am. Heart J..

[36] Tower-Rader A., Jaber W.A. (2019). Multimodality Imaging Assessment of Fabry Disease. Circ. Cardiovasc. Imaging.

[37] Hazari H., Belenkie I., Kryski A., White J.A., Oudit G.Y., Thompson R., Fung T., Dehar N., Khan A. (2018). Comparison of Cardiac Magnetic Resonance Imaging and Echocardiography in Assessment of Left Ventricular Hypertrophy in Fabry Disease. Can. J. Cardiol..

[38] Kozor R., Grieve S.M., Tchan M.C., Callaghan F., Hamilton-Craig C., Denaro C., Moon J.C., A Figtree G. (2016). Cardiac involvement in genotype-positive Fabry disease patients assessed by cardiovascular MR. Heart.

[39] Satriano A., Afzal Y., Afzal M.S., Hassanabad A.F., Wu C., Dykstra S., Flewitt J., Feuchter P., Sandonato R., Heydari B. (2020). Neural-Network-Based Diagnosis Using 3-Dimensional Myocardial Architecture and Deformation: Demonstration for the Differentiation of Hypertrophic Cardiomyopathy. Front. Cardiovasc. Med..

[40] Tanaka H., Adachi K., Yamashita Y., Toshima H., Koga Y. (1988). Four cases of Fabry’s disease mimicking hypertrophic cardio-myopathy. J. Cardiol..

[41] Cianciulli T.F., Saccheri M.C., Fernández S.P., Fernández C.C., Rozenfeld P.A., Kisinovsky I. (2015). Apical Left Ventricular Hypertrophy and Mid-Ventricular Obstruction in Fabry Disease. Echocardiography.

[42] Pieroni M., Chimenti C., De Cobelli F., Morgante E., Del Maschio A., Gaudio C., Russo M.A., Frustaci A. (2006). Fabry’s Disease Cardiomyopathy: Echocardiographic detection of endomyocardial glycosphingolipid compartmentalization. J. Am. Coll. Cardiol..

[43] Mundigler G., Gaggl M., Heinze G., Graf S., Zehetgruber M., Lajic N., Voigtländer T., Mannhalter C., Sunder-Plassmann R., Paschke E. (2011). The endocardial binary appearance (’binary sign’) is an unreliable marker for echocardiographic detection of Fabry disease in patients with left ventricular hypertrophy. Eur. J. Echocardiogr..

[44] Niemann M., Liu D., Hu K., Herrmann S., Breunig F., Strotmann J., Störk S., Voelker W., Ertl G., Wanner C. (2011). Prominent Papillary Muscles in Fabry Disease: A Diagnostic Marker?. Ultrasound Med. Biol..

[45] Hoigné P., Jost C.A., Duru F., Oechslin E., Seifert B., Widmer U., Frischknecht B., Jenni R. (2006). Simple criteria for differentiation of Fabry disease from amyloid heart disease and other causes of left ventricular hypertrophy. Int. J. Cardiol..

[46] Kozor R., Callaghan F., Tchan M., Hamilton-Craig C., A Figtree G., Grieve S.M. (2015). A disproportionate contribution of papillary muscles and trabeculations to total left ventricular mass makes choice of cardiovascular magnetic resonance analysis technique critical in Fabry disease. J. Cardiovasc. Magn. Reson..

[47] Al-Arnawoot A., O’Brien C., Karur G.R., Nguyen E.T., Wasim S., Iwanochko R.M., Morel C.F., Hanneman K. (2020). Clinical Significance of Papillary Muscles on Left Ventricular Mass Quantification Using Cardiac Magnetic Resonance Imaging: Reproducibility and Prognostic Value in Fabry Disease. J. Thorac. Imaging.

[48] Moon J.C., Sachdev B., Elkington A.G., McKenna W.J., Mehta A., Pennell D.J., Leed P.J., Elliott P.M. (2003). Gadolinium enhanced car-diovascular magnetic resonance in Anderson-Fabry disease. Evidence for a disease specific abnormality of the myocardi-al interstitium. Eur. Heart J..

[49] Moon J.C., Sheppard M., Reed E., Lee P., Elliott P.M., Pennell D.J. (2006). The Histological Basis of Late Gadolinium Enhancement Cardiovascular Magnetic Resonance in a Patient with Anderson-Fabry Disease. J. Cardiovasc. Magn. Reson..

[50] Deva D.P., Hanneman K., Li Q., Ng M.Y., Wasim S., Morel C., Iwanochko R.M., Thavendiranathan P., Crean A.M. (2016). Cardiovascular magnetic resonance demonstration of the spectrum of morphological phenotypes and patterns of myocardial scarring in Anderson-Fabry disease. J. Cardiovasc. Magn. Reson..

[51] Elliott P.M., Anastasakis A., Borger M.A., Borggrefe M., Cecchi F., Charron P., Hagege A.A., Lafont A., Limongelli G., Authors/Task Force Members (2014). 2014 ESC Guidelines on diagnosis and management of hypertrophic cardiomyopathy: The Task Force for the diagnosis and management of hypertrophic cardiomyopathy of the European Society of Cardiology (ESC). Eur. Heart J..

[52] Park J.-H., Kwon D.H., Starling R.C., Marwick T.H. (2013). Role of Imaging in the Detection of Reversible Cardiomyopathy. J. Cardiovasc. Ultrasound.

[53] Niemann M., Herrmann S., Hu K., Breunig F., Strotmann J., Beer M., Machann W., Voelker W., Ertl G., Wanner C. (2011). Differences in Fabry Cardiomyopathy Between Female and Male Patients: Consequences for diagnostic assessment. JACC Cardiovasc. Imaging.

[54] Moonen A., Lal S., Ingles J., Yeates L., Semsarian C., Puranik R. (2020). Prevalence of Anderson-Fabry disease in a cohort with unexplained late gadolinium enhancement on cardiac MRI. Int. J. Cardiol..

[55] Weidemann F., Niemann M., Herrmann S., Kung M., Störk S., Waller C., Beer M., Breunig F., Wanner C., Voelker W. (2007). A new echocardiographic approach for the detection of non-ischaemic fibrosis in hypertrophic myocardium. Eur. Heart J..

[56] Krämer J., Niemann M., Liu D., Hu K., Machann W., Beer M., Wanner C., Ertl G., Weidemann F. (2013). Two-dimensional speckle tracking as a non-invasive tool for identification of myocardial fibrosis in Fabry disease. Eur. Heart J..

[57] Niemann M., Hartmann T., Namdar M., Breunig F., Beer M., Machann W., Herrmann S., Ertl G., Wanner C., Weidemann F. (2012). Cross-sectional baseline analysis of electrocardiography in a large cohort of patients with untreated Fabry disease. J. Inherit. Metab. Dis..

[58] Nordin S., Kozor R., Bulluck H., Castelletti S., Rosmini S., Abdel-Gadir A., Baig S., Mehta A., Hughes D., Moon J.C. (2016). Cardiac Fabry Disease With Late Gadolinium Enhancement Is a Chronic Inflammatory Cardiomyopathy. J. Am. Coll. Cardiol..

[59] Seydelmann N., Liu D., Krämer J., Drechsler C., Hu K., Nordbeck P., Schneider A., Störk S., Bijnens B., Ertl G. (2016). High-Sensitivity Troponin: A Clinical Blood Biomarker for Staging Cardiomyopathy in Fabry Disease. J. Am. Heart Assoc..

[60] Yogasundaram H., Nikhanj A., Putko B.N., Boutin M., Jain-Ghai S., Khan A., Auray-Blais C., West M.L., Oudit G.Y. (2018). Elevated Inflammatory Plasma Biomarkers in Patients With Fabry Disease: A Critical Link to Heart Failure With Preserved Ejection Fraction. J. Am. Heart Assoc..

[61] Augusto J.B., Nordin S., Vijapurapu R., Baig S., Bulluck H., Castelletti S., Alfarih M., Knott K., Captur G., Kotecha T. (2020). Myocardial Edema, Myocyte Injury, and Disease Severity in Fabry Disease. Circ. Cardiovasc. Imaging.

[62] Nappi C., Altiero M., Imbriaco M., Nicolai E., Giudice C.A., Aiello M., Diomiaiuti C.T., Pisani A., Spinelli L., Cuocolo A. (2015). First experience of simultaneous PET/MRI for the early detection of cardiac involvement in patients with Anderson-Fabry disease. Eur. J. Nucl. Med. Mol. Imaging.

[63] Imbriaco M., Nappi C., Ponsiglione A., Pisani A., Dell’Aversana S., Nicolai E., Spinelli L., Aiello M., Diomiaiuti C.T., Riccio E. (2019). Hybrid positron emission tomography-magnetic resonance imaging for assessing different stages of cardiac impairment in patients with Anderson–Fabry disease: AFFINITY study group. Eur. Heart J. Cardiovasc. Imaging.

[64] Spinelli L., Imbriaco M., Nappi C., Nicolai E., Giugliano G., Ponsiglione A., Diomiaiuti T.C., Riccio E., Duro G., Pisani A. (2018). Early Cardiac Involvement Affects Left Ventricular Longitudinal Function in Females Carrying α-Galactosidase A Mutation: Role of Hybrid Positron Emission Tomography and Magnetic Resonance Imaging and Speck-le-Tracking Echocardiography. Circ. Cardiovasc. Imaging.

[65] Weidemann F., Niemann M., Breunig F., Herrmann S., Beer M., Störk S., Voelker W., Ertl G., Wanner C., Strotmann J. (2009). Long-Term Effects of Enzyme Replacement Therapy on Fabry Cardiomyopathy: Evidence for a better outcome with early treatment. Circulation.

[66] Krämer J., Niemann M., Störk S., Frantz S., Beer M., Ertl G., Wanner C., Weidemann F. (2014). Relation of Burden of Myocardial Fibrosis to Malignant Ventricular Arrhythmias and Outcomes in Fabry Disease. Am. J. Cardiol..

[67] Baig S., Edward N.C., Kotecha D., Liu B., Nordin S., Kozor R., Moon J.C., Geberhiwot T., Steeds R.P. (2017). Ventricular arrhythmia and sudden cardiac death in Fabry disease: A systematic review of risk factors in clinical practice. Europace.

[68] Sado D.M., White S.K., Piechnik S.K., Banypersad S.M., Treibel T., Captur G., Fontana M., Maestrini V., Flett A.S., Robson M.D. (2013). Identification and Assessment of Anderson-Fabry Disease by Cardiovascular Magnetic Resonance Noncontrast Myocardial T1 Mapping. Circ. Cardiovasc. Imaging.

[69] Nordin S., Kozor R., Baig S., Abdel-Gadir A., Medina-Menacho K., Rosmini S., Captur G., Tchan M., Geberhiwot T., Murphy E. (2018). Cardiac Phenotype of Prehypertrophic Fabry Disease. Circ. Cardiovasc. Imaging.

[70] Camporeale A., Pieroni M., Pieruzzi F., Lusardi P., Pica S., Spada M., Mignani R., Burlina A., Bandera F., Guazzi M. (2019). Predictors of Clinical Evolution in Prehypertrophic Fabry Disease. Circ. Cardiovasc. Imaging.

[71] Pica S., Sado D.M., Maestrini V., Fontana M., White S.K., Treibel T., Captur G., Anderson S., Piechnik S.K., Robson M.D. (2014). Reproducibility of native myocardial T1 mapping in the assessment of Fabry disease and its role in early detection of cardiac involvement by cardiovascular magnetic resonance. J. Cardiovasc. Magn. Reson..

[72] Vijapurapu R., Nordin S., Baig S., Liu B., Rosmini S., Augusto J., Tchan M., A Hughes D., Geberhiwot T., Moon J.C. (2018). Global longitudinal strain, myocardial storage and hypertrophy in Fabry disease. Heart.

[73] Messroghli D.R., Moon J.C., Ferreira V.M., Grosse-Wortmann L., He T., Kellman P., Mascherbauer J., Nezafat R., Salerno M., Schelbert E.B. (2017). Clinical recommendations for cardiovascular magnetic resonance mapping of T1, T2, T2* and extracellular volume: A consensus statement by the Society for Cardiovascular Magnetic Resonance (SCMR) endorsed by the European Association for Cardiovascular Imaging (EACVI). J. Cardiovasc. Magn. Reson..

[74] Pagano J.J., Chow K., Khan A., Michelakis E., Paterson I., Oudit G.Y., Thompson R.B. (2016). Reduced Right Ventricular Native Myocardial T1 in Anderson-Fabry Disease: Comparison to Pulmonary Hypertension and Healthy Controls. PLoS ONE.

[75] Nordin S., Kozor R., Medina-Menacho K., Abdel-Gadir A., Baig S., Sado D.M., Lobascio I., Murphy E., Lachmann R.H., Mehta A. (2019). Proposed Stages of Myocardial Phenotype Development in Fabry Disease. JACC: Cardiovasc. Imaging.

[76] Kawano M., Takenaka T., Otsuji Y., Teraguchi H., Yoshifuku S., Yuasa T., Yu B., Miyata M., Hamasaki S., Minagoe S. (2007). Significance of Asymmetric Basal Posterior Wall Thinning in Patients With Cardiac Fabry’s Disease. Am. J. Cardiol..

[77] Niemann M., Breunig F., Beer M., Hu K., Liu D., Emmert A., Herrmann S., Ertl G., Wanner C., Takenaka T. (2011). Tei Index in Fabry Disease. J. Am. Soc. Echocardiogr..

[78] Liu D., Oder D., Salinger T., Hu K., Müntze J., Weidemann F., Herrmann S., Ertl G., Wanner C., Frantz S. (2018). Association and diagnostic utility of diastolic dysfunction and myocardial fibrosis in patients with Fabry disease. Open Heart.

[79] Torralba-Cabeza M.-Á., Olivera S., Hughes D.A., Pastores G.M., Mateo R.N., Pérez-Calvo J.-I. (2011). Cystatin C and NT-proBNP as prognostic biomarkers in Fabry disease. Mol. Genet. Metab..

[80] Wu J.C., Ho C.Y., Skali H., Abichandani R., Wilcox W.R., Banikazemi M., Packman S., Sims K., Solomon S.D. (2010). Cardiovascular manifestations of Fabry disease: Relationships between left ventricular hypertrophy, disease severity, and alpha-galactosidase A activity. Eur. Heart J..

[81] Pieroni M., Chimenti C., Ricci R., Sale P., Russo M.A., Frustaci A. (2003). Early Detection of Fabry Cardiomyopathy by Tissue Doppler Imaging. Circulation.

[82] Saccheri M.C., Cianciulli T.F., Lax J.A., Gagliardi J.A., Cáceres G.L., Quarin A.E., Kisinovsky I., Rozenfeld P.A., Reisin R.C. (2013). Aadelfa Two-Dimensional Speckle Tracking Echocardiography for Early Detection of Myocardial Damage in Young Patients with Fabry Disease. Echocardiography.

[83] Spinelli L., Giugliano G., Imbriaco M., Esposito G., Nappi C., Riccio E., Ponsiglione A., Pisani A., Cuocolo A., Trimarco B. (2020). Left ventricular radial strain impairment precedes hypertrophy in Anderson–Fabry disease. Int. J. Cardiovasc. Imaging.

[84] Zada M., Lo Q., Boyd A.C., Bradley S., Devine K., Denaro C.P., Sadick N., Richards D.A., Tchan M.C., Thomas L. (2020). Basal Segmental Longitudinal Strain: A Marker of Subclinical Myocardial Involvement in Anderson-Fabry Disease. J. Am. Soc. Echocardiogr..

[85] Shanks M., Thompson R.B., Paterson I.D., Putko B., Khan A., Chan A., Becher H., Oudit G.Y. (2013). Systolic and Diastolic Function Assessment in Fabry Disease Patients Using Speckle-Tracking Imaging and Comparison with Conventional Echocardiographic Measurements. J. Am. Soc. Echocardiogr..

[86] Cianciulli T.F., Saccheri M.C., Rísolo M.A., Lax J.A., Méndez R.J., Morita L.A., Beck M.A., Kazelián L.R. (2020). Mechanical dispersion in Fabry disease assessed with speckle tracking echocardiography. Echocardiography.

[87] Réant P., Testet E., Reynaud A., Bourque C., Michaud M., Rooryck C., Goizet C., Lacombe D., De-Précigout V., Peyrou J. (2020). Characterization of Fabry Disease cardiac involvement according to longitudinal strain, cardiometabolic exercise test, and T1 mapping. Int. J. Cardiovasc. Imaging.

[88] Gruner C., Verocai F., Carasso S., Vannan M.A., Jamorski M., Clarke J.T., Care M., Iwanochko R.M., Rakowski H. (2012). Systolic Myocardial Mechanics in Patients with Anderson-Fabry Disease with and without Left Ventricular Hypertrophy and in Comparison to Nonobstructive Hypertrophic Cardiomyopathy. Echocardiography.

[89] Graziani F., Lillo R., Panaioli E., Spagnoletti G., Pieroni M., Ferrazzi P., Camporeale A., Verrecchia E., Sicignano L.L., Manna R. (2021). Evidence of evolution towards left midventricular obstruction in severe Anderson–Fabry cardiomyopathy. ESC Heart Fail..

[90] Calcagnino M., O’Mahony C., Coats C., Cardona M., Garcia A., Janagarajan K., Mehta A., Hughes D., Murphy E., Lachmann R. (2011). Exercise-Induced Left Ventricular Outflow Tract Obstruction in Symptomatic Patients with Anderson-Fabry Disease. J. Am. Coll. Cardiol..

[91] Spinelli L., Nicolai E., Acampa W., Imbriaco M., Pisani A., Rao M.A.E., Scopacasa F., Cianciaruso B., De Luca N., Cuocolo A. (2008). Cardiac performance during exercise in patients with Fabry’s disease. Eur. J. Clin. Investig..

[92] Niemann M., Breunig F., Beer M., Herrmann S., Strotmann J., Hu K., Emmert A., Voelker W., Ertl G., Wanner C. (2010). The right ventricle in Fabry disease: Natural history and impact of enzyme replacement therapy. Heart.

[93] Graziani F., Laurito M., Pieroni M., Pennestrì F., Lanza G.A., Coluccia V., Camporeale A., Pedicino D., Verrecchia E., Manna R. (2017). Right Ventricular Hypertrophy, Systolic Function, and Disease Severity in Anderson-Fabry Disease: An Echocardiographic Study. J. Am. Soc. Echocardiogr..

[94] Morris D.A., Blaschke D., Canaan-Kühl S., Krebs A., Knobloch G., Walter T.C., Haverkamp W. (2014). Global cardiac alterations detected by speckle-tracking echocardiography in Fabry disease: Left ventricular, right ventricular, and left atrial dysfunction are common and linked to worse symptomatic status. Int. J. Cardiovasc. Imaging.

[95] Graziani F., Lillo R., Panaioli E., Pieroni M., Camporeale A., Verrecchia E., Sicignano L.L., Manna R., Lombardo A., Lanza G.A. (2020). Prognostic significance of right ventricular hypertrophy and systolic function in Anderson–Fabry disease. ESC Heart Fail..

[96] Boyd A.C., Lo Q., Devine K., Tchan M.C., Sillence D.O., Sadick N., Richards D.A., Thomas L. (2013). Left Atrial Enlargement and Reduced Atrial Compliance Occurs Early in Fabry Cardiomyopathy. J. Am. Soc. Echocardiogr..

[97] Esposito R., Russo C., Santoro C., Cocozza S., Riccio E., Sorrentino R., Pontillo G., Luciano F., Imbriaco M., Brunetti A. (2020). Association between Left Atrial Deformation and Brain Involvement in Patients with Anderson-Fabry Disease at Diagnosis. J. Clin. Med..

[98] Shah J.S., Hughes D.A., Sachdev B., Tome M., Ward D., Lee P., Mehta A.B., Elliott P.M. (2005). Prevalence and Clinical Significance of Cardiac Arrhythmia in Anderson-Fabry Disease. Am. J. Cardiol..

[99] Coats C.J., Parisi V., Ramos M., Janagarajan K., O’Mahony C., Dawnay A., Lachmann R.H., Murphy E., Mehta A., Hughes D. (2013). Role of Serum N-Terminal Pro-Brain Natriuretic Peptide Measurement in Diagnosis of Cardiac Involvement in Patients With Anderson-Fabry Disease. Am. J. Cardiol..

[100] Patel V., O’Mahony C., Hughes D., Rahman M.S., Coats C., Murphy E., Lachmann R., Mehta A., Elliott P.M. (2015). Clinical and genetic predictors of major cardiac events in patients with Anderson–Fabry Disease. Heart.

[101] Kampmann C., Wiethoff C.M., Whybra C., Baehner F.A., Mengel E., Beck M. (2008). Cardiac manifestations of Anderson-Fabry disease in children and adolescents. Acta Paediatr..

[102] Frustaci A., Morgante E., Russo M.A., Scopelliti F., Grande C., Verardo R., Franciosa P., Chimenti C. (2015). Pathology and Function of Conduction Tissue in Fabry Disease Cardiomyopathy. Circ. Arrhythmia Electrophysiol..

[103] Roudebush C.P., Foerster J.M., Bing O.H.L. (1973). The Abbreviated Pr Interval of Fabry’s Disease. N. Engl. J. Med..

[104] Omar A.R., Harris L., Cameron D.A., Chauhan V.S. (2006). WPW and Fabry’s disease: Evidence for atrioventricular and atriohisian acces-sory pathway conduction. HeartRhythm.

[105] Jastrzebski M., Bacior B., Dimitrow P.P., Kawecka-Jaszcz K. (2006). Electrophysiological study in a patient with Fabry disease and a short PQ interval. Europace.

[106] Waldek S. (2003). PR Interval and the Response to Enzyme-Replacement Therapy for Fabry’s Disease. N. Engl. J. Med..

[107] Wolf C.M., Arad M., Ahmad F., Sanbe A., Bernstein S.A., Toka O., Konno T., Morley G., Robbins J., Seidman J. (2008). Reversibility of PRKAG2 Glycogen-Storage Cardiomyopathy and Electrophysiological Manifestations. Circulation.

[108] Namdar M., Kampmann C., Steffel J., Walder D., Holzmeister J., Lüscher T.F., Jenni R., Duru F. (2010). PQ Interval in Patients With Fabry Disease. Am. J. Cardiol..

[109] Mehta J., Tuna N., Moller J.H., Desnick R.J. (1977). Electrocardiographic and Vectorcardiographic Observations in Fabrys Disease. Adv. Cardiol..

[110] Ikari Y., Kuwako K., Yamaguchi T. (1992). Fabry’s disease with complete atrioventricular block: Histological evidence of involvement of the conduction system. Heart.

[111] O’Mahony C., Coats C., Cardona M., Garcia A., Calcagnino M., Murphy E., Lachmann R., Mehta A., Hughes D., Elliott P.M. (2011). Incidence and predictors of anti-bradycardia pacing in patients with Anderson-Fabry disease. Europace.

[112] Lobo T., Morgan J., Bjorksten A., Nicholls K., Grigg L., Centra E., Becker G. (2008). Cardiovascular testing in Fabry disease: Exercise capacity reduction, chronotropic incompetence and improved anaerobic threshold after enzyme replacement. Intern. Med. J..

[113] Powell A.W., Jefferies J.L., Hopkin R.J., Mays W.A., Goa Z., Chin C. (2018). Cardiopulmonary fitness assessment on maximal and submaximal exercise testing in patients with Fabry disease. Am. J. Med. Genet. Part A.

[114] Di L.Z., Pichette M., Nadeau R., Bichet D.G., Poulin F. (2018). Severe bradyarrhythmia linked to left atrial dysfunction in Fabry disease-A cross-sectional study. Clin. Cardiol..

[115] Chimenti C., Russo M.A., Frustaci A. (2010). Atrial biopsy evidence of Fabry disease causing lone atrial fibrillation. Heart.

[116] Pinderski L.J., Strotmann J. (2006). Congestive heart failure in Fabry cardiomyopathy: Natural history experience in an interna-tional cohort of 1448 patients. J. Heart Lung Transpl..

[117] Hanneman K., Karur G.R., Wasim S., Wald R.M., Iwanochko R.M., Morel C.F. (2020). Left Ventricular Hypertrophy and Late Gadolinium Enhancement at Cardiac MRI Are Associated with Adverse Cardiac Events in Fabry Disease. Radiology.

[118] Frustaci A., Chimenti C. (2007). Images in cardiovascular medicine. Cryptogenic Ventricular Arrhythmias and Sudden Death by Fabry Disease: Prominent Infiltration of Cardiac Conduction Tissue. Circulation.

[119] Higashi H., Yamagata K., Noda T., Satomi K. (2011). Endocardial and epicardial substrates of ventricular tachycardia in a patient with Fabry disease. Heart Rhythm..

[120] Weidemann F., Niemann M., Störk S., Breunig F., Beer M., Sommer C., Herrmann S., Ertl G., Wanner C. (2013). Long-term outcome of enzyme-replacement therapy in advanced F abry disease: Evidence for disease progression towards serious complications. J. Intern. Med..

[121] Imbriaco M., Pellegrino T., Piscopo V., Petretta M., Ponsiglione A., Nappi C., Puglia M., Dell’Aversana S., Riccio E., Spinelli L. (2017). Cardiac sympathetic neuronal damage precedes myocardial fibrosis in patients with Anderson-Fabry disease. Eur. J. Nucl. Med. Mol. Imaging.

[122] Weidemann F., Maier S.K., Störk S., Brunner T., Liu D., Hu K., Seydelmann N., Schneider A., Becher J., Canan-Kühl S. (2016). Usefulness of an Implantable Loop Recorder to Detect Clinically Relevant Arrhythmias in Patients with Advanced Fabry Cardiomyopathy. Am. J. Cardiol..

[123] Sené T., Lidove O., Sebbah J., Darondel J.-M., Picard H., Aaron L., Fain O., Zenone T., Joly D., Charron P. (2016). Cardiac device implantation in Fabry disease: A retrospective monocentric study. Medicine.

[124] Vijapurapu R., Geberhiwot T., Jovanovic A., Baig S., Nordin S., Kozor R., Leyva F., Kotecha D., Wheeldon N., Deegan P. (2019). Study of indications for cardiac device implantation and utilisation in Fabry cardiomyopathy. Heart.

[125] Acharya D., Robertson P., Kay G.N., Jackson L., Warnock D.G., Plumb V.J., Tallaj J.A. (2012). Arrhythmias in Fabry Cardiomyopathy. Clin. Cardiol..

[126] Chimenti C., Morgante E., Tanzilli G., Mangieri E., Critelli G., Gaudio C., Russo M.A., Frustaci A. (2008). Angina in Fabry Disease Reflects Coronary Small Vessel Disease. Circ. Heart Fail..

[127] Kalliokoski R.J., Kalliokoski K.K., Sundell J., Engblom E., Penttinen M., Kantola I., Raitakari O.T., Knuuti J., Nuutila P. (2005). Impaired myocardial perfusion reserve but preserved peripheral endothelial function in patients with Fabry disease. J. Inherit. Metab. Dis..

[128] Elliott P.M., Kindler H., Shah J.S., Sachdev B., E Rimoldi O., Thaman R., Tome M.T., McKenna W.J., Lee P., Camici P.G. (2005). Coronary microvascular dysfunction in male patients with Anderson-Fabry disease and the effect of treatment with alphagalactosidase A. Heart.

[129] Tomberli B., Cecchi F., Sciagrà R., Berti V., Lisi F., Torricelli F., Morrone A., Castelli G., Yacoub M.H., Olivotto I. (2013). Coronary microvascular dysfunction is an early feature of cardiac involvement in patients with Anderson-Fabry disease. Eur. J. Heart Fail..

[130] Knott K.D., Augusto J.B., Nordin S., Kozor R., Camaioni C., Xue H., Hughes R.K., Manisty C., Brown L.A., Kellman P. (2019). Quantitative Myocardial Perfusion in Fabry Disease. Circ. Cardiovasc. Imaging.

[131] Kitani Y., Nakagawa N., Sakamoto N., Takeuchi T., Takahashi F., Momosaki K., Nakamura K., Endo F., Maruyama H., Hasebe N. (2019). Unexpectedly High Prevalence of Coronary Spastic Angina in Patients with Anderson-Fabry Disease. Circ. J..

[132] Ogawa T., Kawai M., Matsui T., Seo A., Aizawa O., Hongo K., Shibata T., Yoshida S., Okamura T., Nishikawa T. (1996). Vasospastic Angina in a Patient with Fabry’s Disease Who Showed Normal Coronary Angiographic Findings. Jpn. Circ. J..

[133] Kodama K., Ozawa T., Dochi K., Ueno Y. (2019). Ventricular fibrillation associated with vasospastic angina pectoris in Fabry disease: A case report. Eur. Heart J. Case Rep..

[134] Linhart A., Mehta A., Beck M., Sunder-Plassmann G. (2006). The heart in Fabry disease. Fabry Disease: Perspectives from 5 Years of FOS.

[135] Graziani F., Lillo R., Panaioli E., Spagnoletti G., Bruno I., Leccisotti L., Marano R., Manna R., Crea F. (2019). Massive Coronary Microvascular Dysfunction in Severe Anderson-Fabry Disease Cardiomyopathy. Circ. Cardiovasc. Imaging.

[136] Schiffmann R., Rapkiewicz A., Abu-Asab M., Ries M., Askari H., Tsokos M., Quezado M. (2005). Pathological findings in a patient with Fabry disease who died after 2.5 years of enzyme replacement. Virchows Arch..

[137] Frustaci A., Russo M.A., Francone M., Chimenti C. (2014). Microvascular Angina as Prehypertrophic Presentation of Fabry Disease Cardiomyopathy. Circulation.

[138] Patel M.R., Cecchi F., Cizmarik M., Kantola I., Linhart A., Nicholls K., Strotmann J., Tallaj J., Tran T.C., West M.L. (2011). Cardiovascular Events in Patients With Fabry Disease: Natural history data from the fabry registry. J. Am. Coll. Cardiol..

[139] Hernández-Hernández A., Diez-López C., Azevedo O., Palomino-Doza J., Alfonso F., Fuentes-Cañamero M.E., Jiménez M.V.M., Rodríguez C.C., Ruz A., Tirón C. (2021). Screening of Fabry Disease in Patients with Chest Pain Without Obstructive Coronary Artery Disease. J. Cardiovasc. Transl. Res..

[140] Sakuraba H., Yanagawa Y., Igarashi T., Suzuki Y., Suzuki T., Watanabe K., Ieki K., Shimoda K., Yamanaka T. (1986). Cardiovascular manifestations in Fabry’s disease. A high incidence of mitral valve prolapse in hemizygotes and heterozygotes. Clin. Genet..

[141] Barbey F., Qanadli S.D., Juli C., Brakch N., Palaček T., Rizzo E., Jeanrenaud X., Eckhardt B., Linhart A. (2009). Aortic remodelling in Fabry disease. Eur. Heart J..

[142] Spinelli L., Giugliano G., Pisani A., Imbriaco M., Riccio E., Russo C., Cuocolo A., Trimarco B., Esposito G. (2020). Does left ventricular function predict cardiac outcome in Anderson–Fabry disease?. Int. J. Cardiovasc. Imaging.

[143] Talbot A.S., Lewis N.T., Nicholls K.M. (2014). Cardiovascular outcomes in Fabry disease are linked to severity of chronic kidney disease. Heart.

[144] Rosmini S., Biagini E., O’Mahony C., Bulluck H., Ruozi N., Lopes L.R., Guttmann O., Reant P., Quarta C.C., Pantazis A. (2016). Relationship between aetiology and left ventricular systolic dysfunction in hypertrophic cardiomyopathy. Heart.

[145] Linhart A., Germain D.P., Olivotto I., Akhtar M.M., Anastasakis A., Hughes D., Namdar M., Pieroni M., Hagège A., Cecchi F. (2020). An expert consensus document on the management of cardiovascular manifestations of Fabry disease. Eur. J. Heart Fail..

[146] Eng C.M., Ashley G.A., Burgert T.S., Enriquez A.L., D’Souza M., Desnick R.J. (1997). Fabry disease: Thirty-five mutations in the alpha-galactosidase A gene in patients with classic and variant phenotypes. Mol. Med..

[147] Bishop D.F., Grabowski G.A., Desnick R.J. (1981). Fabry disease: An asymptomatic hemizygote with significant residual a-galactosidase activity. Am. J. Hum. Genet..

[148] Eng C.M., Resnick-Silverman L.A., Niehaus D.J., Astrin K.H., Desnick R.J. (1993). Nature and frequency of mutations in the alpha-galactosidase A gene that cause Fabry disease. Am. J. Hum. Genet..

[149] Germain D.P., Brand E., Burlina A., Cecchi F., Garman S.C., Kempf J., Laney D.A., Linhart A., Maródi L., Nicholls K. (2018). Phenotypic characteristics of the p.Asn215Ser (p.N215S) GLA mutation in male and female patients with Fabry disease: A multicenter Fabry Registry study. Mol. Genet. Genom. Med..

[150] LaValle L., Thomas A.S., Beaton B., Ebrahim H., Reed M., Ramaswami U., Elliott P., Mehta A.B., Hughes D.A. (2018). Phenotype and biochemical heterogeneity in late onset Fabry disease defined by N215S mutation. PLoS ONE.

[151] Ishii S., Nakao S., Minamikawa-Tachino R., Desnick R.J., Fan J.-Q. (2002). Alternative Splicing in the alpha-Galactosidase A Gene: Increased Exon Inclusion Results in the Fabry Cardiac Phenotype. Am. J. Hum. Genet..

[152] Nakao S., Takenaka T., Maeda M., Kodama C., Tanaka A., Tahara M., Yoshida A., Kuriyama M., Hayashibe H., Sakuraba H. (1995). An Atypical Variant of Fabry’s Disease in Men with Left Ventricular Hypertrophy. N. Engl. J. Med..

[153] Visoiu I.-S., Ciobanu A.O., Nicula A.I., Iascone M., Jurcut R., Vinereanu D., Rimbas R.C. (2019). Severe Late-Onset Fabry Cardiomyopathy Unmasked by a Multimodality Imaging Approach. Circ. Cardiovasc. Imaging.

[154] Adalsteinsdottir B., Palsson R., Desnick R.J., Gardarsdottir M., Teekakirikul P., Maron M., Appelbaum E., Neisius U., Maron B.J., Burke M.A. (2017). Fabry Disease in Families with Hypertrophic Cardiomyopathy: Clinical Manifestations in the Clas-sic and Later-Onset Phenotypes. Circ. Cardiovasc. Genet..

[155] Csányi B., Hategan L., Nagy V., Obál I., Varga E.T., Borbás J., Tringer A., Eichler S., Forster T., Rolfs A. (2017). Identification of a Novel GLA Gene Mutation, p.Ile239Met, in Fabry Disease With a Predominant Cardiac Phenotype. Int. Heart J..

[156] Ishii S., Kase R., Sakuraba H., Suzuki Y. (1993). Characterization of a Mutant alpha-Galactosidase Gene Product for the Late-Onset Cardiac Form of Fabry Disease. Biochem. Biophys. Res. Commun..

[157] Kase R., Bierfreund U., Klein A., Kolter T., Utsumi K., Itoh K., Sandhoff K., Sakuraba H. (2000). Characterization of two alpha-galactosidase mutants (Q279E and R301Q) found in an atypical variant of Fabry disease. Biochim. Biophys. Acta Mol. Basis Dis..

[158] Yoshitama T., Nakao S., Takenaka T., Teraguchi H., Sasaki T., Kodama C., Tanaka A., Kisanuki A., Tei C. (2001). Molecular genetic, biochemical, and clinical studies in three families with cardiac Fabry’s disease. Am. J. Cardiol..

[159] Von Scheidt W., Eng C.M., Fitzmaurice T.F., Erdmann E., Hübner G., Olsen E.G., Christomanou H., Kandolf R., Bishop D.F., Desnick R.J. (1991). An Atypical Variant of Fabry’s Disease with Manifestations Confined to the Myocardium. N. Engl. J. Med..

[160] Sakuraba H., Oshima A., Fukuhara Y., Shimmoto M., Nagao Y., Bishop D.F., Desnick R.J., Suzuki Y. (1990). Identification of point mutations in the alpha-galactosidase A gene in classical and atypical hemizygotes with Fabry disease. Am. J. Hum. Genet..

[161] Frustaci A., Chimenti C., Ricci R., Natale L., Russo M.A., Pieroni M., Eng C.M., Desnick R.J. (2001). Improvement in Cardiac Function in the Cardiac Variant of Fabry’s Disease with Galactose-Infusion Therapy. N. Engl. J. Med..

[162] Liang K.-H., Lu Y.-H., Niu C.-W., Chang S.-K., Chen Y.-R., Cheng C.-Y., Hsu T.-R., Yang C.-F., Nakamura K., Niu D.-M. (2020). The Fabry disease-causing mutation, GLA IVS4+919G>A, originated in Mainland China more than 800 years ago. J. Hum. Genet..

[163] Lin H.-Y., Huang C.-H., Yu H.-C., Chong K.-W., Hsu J.-H., Lee P.-C., Cheng K.-H., Chiang C.-C., Ho H.-J., Lin S.-P. (2010). Enzyme assay and clinical assessment in subjects with a Chinese hotspot late-onset Fabry mutation (IVS4 + 919G→A). J. Inherit. Metab. Dis..

[164] Oder D., Liu D., Hu K., Üçeyler N., Salinger T., Müntze J., Lorenz K., Kandolf R., Gröne H.-J., Sommer C. (2017). α-Galactosidase A Genotype N215S Induces a Specific Cardiac Variant of Fabry Disease. Circ. Cardiovasc. Genet..

[165] Alharbi F.J., Baig S., Auray-Blais C., Boutin M., Ward D.G., Wheeldon N., Steed R., Dawson C., Hughes D., Geberhiwot T. (2018). Globotriaosylsphingosine (Lyso-Gb3) as a biomarker for cardiac variant (N215S) Fabry disease. J. Inherit. Metab. Dis..

[166] Lin H.-Y., Liu H.-C., Huang Y.-H., Liao H.-C., Hsu T.-R., Shen C.-I., Li S.-T., Li C.-F., Lee L.-H., Lee P.-C. (2013). Effects of enzyme replacement therapy for cardiac-type Fabry patients with a Chinese hotspot late-onset Fabry mutation (IVS4+919G>A). BMJ Open.

[167] Liu H.-C., Lin H.-Y., Yang C.-F., Liao H.-C., Hsu T.-R., Lo C.-W., Chang F.-P., Huang C.-K., Lu Y.-H., Lin S.-P. (2014). Globotriaosylsphingosine (lyso-Gb3) might not be a reliable marker for monitoring the long-term therapeutic outcomes of enzyme replacement therapy for late-onset Fabry patients with the Chinese hotspot mutation (IVS4+919G>A). Orphanet J. Rare Dis..

[168] Chien Y., Chien C.-S., Chiang H.-C., Huang W.-L., Chou S.-J., Chang W.-C., Chang Y.-L., Leu H.-B., Chen K.-H., Wang K.-L. (2016). Interleukin-18 deteriorates Fabry cardiomyopathy and contributes to the development of left ventricular hypertrophy in Fabry patients with GLA IVS4+919 G>A mutation. Oncotarget.

[169] Auray-Blais C., Lavoie P., Boutin M., Ntwari A., Hsu T.-R., Huang C.-K., Niu D.-M. (2017). Biomarkers associated with clinical manifestations in Fabry disease patients with a late-onset cardiac variant mutation. Clin. Chim. Acta.

[170] Hsu T.-R., Sung S.-H., Chang F.-P., Yang C.-F., Liu H.-C., Lin H.-Y., Huang C.-K., Gao H.-J., Huang Y.-H., Liao H.-C. (2014). Endomyocardial biopsies in patients with left ventricular hypertrophy and a common Chinese later-onset fabry mutation (IVS4 + 919G > A). Orphanet J. Rare Dis..

[171] Hsu T.-R., Hung S.-C., Chang F.-P., Yu W.-C., Sung S.-H., Hsu C.-L., Dzhagalov I., Yang C.-F., Chu T.-H., Lee H.-J. (2016). Later Onset Fabry Disease, Cardiac Damage Progress in Silence: Experience with a Highly Prevalent Mutation. J. Am. Coll. Cardiol..

[172] Wang W.-T., Sung S.-H., Liao J.-N., Hsu T.-R., Niu D.-M., Yu W.-C. (2020). Cardiac manifestations in patients with classical or cardiac subtype of Fabry disease. J. Chin. Med. Assoc..

[173] Lee H.-J., Hsu T.-R., Hung S.-C., Yu W.-C., Chu T.-H., Yang C.-F., Bizjajeva S., Tiu C.-M., Niu D.-M. (2017). A comparison of central nervous system involvement in patients with classical Fabry disease or the later-onset subtype with the IVS4+919G>A mutation. BMC Neurol..

[174] Lee H.-J., Hung S.-C., Hsu T.-R., Ko S.-C., Chui-Mei T., Huang C.-C., Niu D.-M., Lin C.-P. (2016). Brain MR Imaging Findings of Cardiac-Type Fabry Disease with an IVS4+919G>A Mutation. Am. J. Neuroradiol..

[175] Azevedo O., Gago M.F., Miltenberger-Miltenyi G., Sousa N., Cunha D. (2020). Fabry Disease Therapy: State-of-the-Art and Current Challenges. Int. J. Mol. Sci..

[176] Ortiz A., Germain D.P., Desnick R.J., Politei J., Mauer M., Burlina A., Eng C., Hopkin R.J., Laney D., Linhart A. (2018). Fabry disease revisited: Management and treatment recommendations for adult patients. Mol. Genet. Metab..

[177] A Hughes D., Elliott P.M., Shah J., Zuckerman J., Coghlan G., Brookes J., Mehta A.B. (2008). Effects of enzyme replacement therapy on the cardiomyopathy of Anderson Fabry disease: A randomised, double-blind, placebo-controlled clinical trial of agalsidase alfa. Heart.

[178] Hughes D.A., Romero M.Á.B., Hollak C.E., Giugliani R., Deegan P.B. (2011). Response of women with Fabry disease to enzyme replacement therapy: Comparison with men, using data from FOS—The Fabry Outcome Survey. Mol. Genet. Metab..

[179] Kampmann C., Perrin A., Beck M. (2015). Effectiveness of agalsidase alfa enzyme replacement in Fabry disease: Cardiac outcomes after 10 years’ treatment. Orphanet J. Rare Dis..

[180] Baehner F., Kampmann C., Whybra C., Miebach E., Wiethoff C.M., Beck M. (2003). Enzyme replacement therapy in heterozygous females with Fabry disease: Results of a phase IIIB study. J. Inherit. Metab. Dis..

[181] Whybra C., Miebach E., Mengel E., Gal A., Baron K., Beck M., Kampmann C. (2009). A 4-year study of the efficacy and tolerability of enzyme replacement therapy with agalsidase alfa in 36 women with Fabry disease. Genet. Med..

[182] Beck M., Hughes D., Kampmann C., Larroque S., Mehta A., Pintos-Morell G., Ramaswami U., West M., Wijatyk A., Giugliani R. (2015). Long-term effectiveness of agalsidase alfa enzyme replacement in Fabry disease: A Fabry Outcome Survey analysis. Mol. Genet. Metab. Rep..

[183] Kim J.H., Lee B.H., Cho J.H., Kang E., Choi J.-H., Kim G.-H., Yoo H.-W. (2016). Long-term enzyme replacement therapy for Fabry disease: Efficacy and unmet needs in cardiac and renal outcomes. J. Hum. Genet..

[184] Germain D.P., Weidemann F., Abiose A., Patel M.R., Cizmarik M., Cole J.A., Beitner-Johnson D., Benistan K., Cabrera G., Charrow J. (2013). Analysis of left ventricular mass in untreated men and in men treated with agalsidase-β: Data from the Fabry Registry. Genet. Med..

[185] Motwani M., Banypersad S., Woolfson P., Waldek S. (2012). Enzyme replacement therapy improves cardiac features and severity of Fabry disease. Mol. Genet. Metab..

[186] Thurberg B.L., Fallon J.T., Mitchell R., Aretz T., Gordon R.E., O’Callaghan M.W. (2009). Cardiac Microvascular Pathology in Fabry Disease: Evaluation of endomyocardial biopsies before and after enzyme replacement therapy. Circulation.

[187] Banikazemi M., Bultas J., Waldek S., Wilcox W.R., Whitley C.B., McDonald M., Finkel R., Packman S., Bichet D.G., Warnock D.G. (2007). Agalsidase-Beta Therapy for Advanced Fabry Disease. Ann. Intern. Med..

[188] Ortiz A., Abiose A., Bichet D.G., Cabrera G., Charrow J., Germain D.P., Hopkin R.J., Jovanovic A., Linhart A., Maruti S.S. (2016). Time to treatment benefit for adult patients with Fabry disease receiving agalsidase β: Data from the Fabry Registry. J. Med. Genet..

[189] Hsu T.-R., Chang F.-P., Chu T.-H., Sung S.-H., Bizjajeva S., Yu W.-C., Niu D.-M. (2017). Correlations between Endomyocardial Biopsies and Cardiac Manifestations in Taiwanese Patients with the Chinese Hotspot IVS4+919G>A Mutation: Data from the Fabry Outcome Survey. Int. J. Mol. Sci..

[190] Germain D.P., Charrow J., Desnick R.J., Guffon N., Kempf J., Lachmann R.H., Lemay R., Linthorst G.E., Packman S., Scott C.R. (2015). Ten-year outcome of enzyme replacement therapy with agalsidase beta in patients with Fabry disease. J. Med. Genet..

[191] Feriozzi S., Linhart A., Ramaswami U., Kalampoki V., Gurevich A., Hughes D., Fabry Outcome Survey Study Group (2020). Effects of Baseline Left Ventricular Hypertrophy and Decreased Renal Function on Cardiovascular and Renal Outcomes in Patients with Fabry Disease Treated with Agalsidase Alfa: A Fabry Outcome Survey Study. Clin. Ther..

[192] Arends M., Biegstraaten M., Wanner C., Sirrs S., Mehta A., Elliott P.M., Oder D., Watkinson O.T., Bichet D.G., Khan A. (2018). Agalsidase alfa versus agalsidase beta for the treatment of Fabry disease: An international cohort study. J. Med. Genet..

[193] Bénichou B., Goyal S., Sung C., Norfleet A.M., O’Brien F. (2009). A retrospective analysis of the potential impact of IgG antibodies to agalsidase beta on efficacy during enzyme replacement therapy for Fabry disease. Mol. Genet. Metab..

[194] Lenders M., Stypmann J., Duning T., Schmitz B., Brand S.-M., Brand E. (2015). Serum-Mediated Inhibition of Enzyme Replacement Therapy in Fabry Disease. J. Am. Soc. Nephrol..

[195] van der Veen S., van Kuilenburg A., Hollak C., Kaijen P., Voorberg J., Langeveld M. (2019). Antibodies against recombinant alpha-galactosidase A in Fabry disease: Subclass analysis and impact on response to treatment. Mol. Genet. Metab..

[196] European Medicines Agency (2016). https://www.ema.europa.eu/en/documents/product-information/galafold-epar-product-information_en.pdf.

[197] Germain D.P., Hughes D.A., Nicholls K., Bichet D.G., Giugliani R., Wilcox W.R., Feliciani C., Shankar S.P., Ezgu F., Amartino H. (2016). Treatment of Fabry’s Disease with the Pharmacologic Chaperone Migalastat. N. Engl. J. Med..

[198] Hughes D.A., Nicholls K., Shankar S.P., Sunder-Plassmann G., Koeller D., Nedd K., Vockley G., Hamazaki T., Lachmann R., Ohashi T. (2017). Oral pharmacological chaperone migalastat compared with enzyme replacement therapy in Fabry disease: 18-month results from the randomised phase III ATTRACT study. J. Med. Genet..

[199] Feldt-Rasmussen U., Hughes D., Sunder-Plassmann G., Shankar S., Nedd K., Olivotto I., Ortiz D., Ohashi T., Hamazaki T., Skuban N. (2020). Long-term efficacy and safety of migalastat treatment in Fabry disease: 30-month results from the open-label extension of the randomized, phase 3 ATTRACT study. Mol. Genet. Metab..

[200] Müntze J., Salinger T., Gensler D., Wanner C., Nordbeck P. (2018). Treatment of hypertrophic cardiomyopathy caused by cardiospecific variants of Fabry disease with chaperone therapy. Eur. Heart J..

[201] Müntze J., Gensler D., Maniuc O., Liu D., Cairns T., Oder D., Hu K., Lorenz K., Frantz S., Wanner C. (2019). Oral Chaperone Therapy Migalastat for Treating Fabry Disease: Enzymatic Response and Serum Biomarker Changes After 1 Year. Clin. Pharmacol. Ther..

[202] Riccio E., Zanfardino M., Ferreri L., Santoro C., Cocozza S., Capuano I., Imbriaco M., Feriozzi S., Pisani A., AFFIINITY Group (2020). Switch from enzyme replacement therapy to oral chaperone migalastat for treating fabry disease: Real-life data. Eur. J. Hum. Genet..

[203] Lenders M., Nordbeck P., Kurschat C., Karabul N., Kaufeld J., Hennermann J.B., Patten M., Cybulla M., Müntze J., Üçeyler N. (2020). Treatment of Fabry’s Disease with Migalastat: Outcome From a Prospective Observational Multicenter Study (FAMOUS). Clin. Pharmacol. Ther..

[204] Kramer J., Bijnens B., Störk S., Ritter C.O., Liu D., Ertl G., Wanner C., Weidemann F. (2015). Left Ventricular Geometry and Blood Pressure as Predictors of Adverse Progression of Fabry Cardiomyopathy. PLoS ONE.

[205] Tahir H., Jackson L.L., Warnock D.G. (2007). Antiproteinuric Therapy and Fabry Nephropathy: Sustained Reduction of Proteinuria in Patients Receiving Enzyme Replacement Therapy with Agalsidase-beta. J. Am. Soc. Nephrol..

[206] Warnock D.G., Thomas C.P., Vujkovac B., Campbell R.C., Charrow J., A Laney D., Jackson L.L., Wilcox W.R., Wanner C. (2015). Antiproteinuric therapy and Fabry nephropathy: Factors associated with preserved kidney function during agalsidase-beta therapy. J. Med. Genet..

[207] Ponikowski P., Voors A.A., Anker S.D., Bueno H., Cleland J.G.F., Coats A.J.S., Falk V., González-Juanatey J.R., Harjola V.-P., Jankowska E.A. (2016). 2016 ESC Guidelines for the diagnosis and treatment of acute and chronic heart failure: The Task Force for the diagnosis and treatment of acute and chronic heart failure of the European Society of Cardiology (ESC)Developed with the special contribution of the Heart Failure Association (HFA) of the ESC. Eur. Heart J..

[208] Knuuti J., Wijns W., Achenbach S., Agewall S., Barbato E., Bax J.J., Capodanno D., Cuisset T., Deaton C., Dickstein K. (2020). 2019 ESC Guidelines for the diagnosis and management of chronic coronary syndromes. Eur. Heart J..

[209] Fukuda Y., Onishi T., Suzuki A., Tanaka H., Fukuzawa K., Yoshida A., Kawai H., Hirata K.-I. (2017). Follow-up of Cardiac Fabry Disease Treated by Cardiac Resynchronization Therapy. CASE.

[210] Meghji Z., Nguyen A., Miranda W.R., Geske J.B., Schaff H.V., Peck D.S., Newman D.B. (2019). Surgical septal myectomy for relief of dynamic obstruction in Anderson-Fabry Disease. Int. J. Cardiol..

[211] Magage S., Linhart A., Bultas J., Vojacek J., Mates M., Palecek T., Popelová J., Tintera J., Aschermann M., Goldman M.E. (2005). Fabry Disease: Percutaneous Transluminal Septal Myocardial Ablation Markedly Improved Symptomatic Left Ventricular Hypertrophy and Outflow Tract Obstruction in a Classically Affected Male. Echocardiography.

[212] Liu D., Hu K., Schmidt M., Müntze J., Maniuc O., Gensler D., Oder D., Salinger T., Weidemann F., Ertl G. (2018). Value of the CHA2DS2-VASc score and Fabry-specific score for predicting new-onset or recurrent stroke/TIA in Fabry disease patients without atrial fibrillation. Clin. Res. Cardiol..

[213] Fine N.M., Wang Y., Khan A. (2019). Acute Decompensated Heart Failure After Initiation of Amiodarone in a Patient With Anderson-Fabry Disease. Can. J. Cardiol..

[214] Halliwell W.H. (1997). Cationic Amphiphilic Drug-Induced Phospholipidosis. Toxicol. Pathol..

[215] Qian P., Ross D., Tchan M., Sadick N., Tchan M. (2015). A patient with recurrent disabling atrial fibrillation and Fabry cardiomyopathy successfully treated with single ring pulmonary vein isolation. Int. J. Cardiol..

[216] Hagège A., Réant P., Habib G., Damy T., Barone-Rochette G., Soulat G., Donal E., Germain D.P. (2019). Fabry disease in cardiology practice: Literature review and expert point of view. Arch. Cardiovasc. Dis..

[217] Cantor W.J., Daly P., Iwanochko M., Clarke J.T., Cusimano R.J., Butany J. (1998). Cardiac transplantation for Fabry’s disease. Can J. Cardiol..

